# Microenvironment Activatable Nanoprodrug Based on Gripper-like Cyclic Phenylboronic Acid to Precisely and Effectively Alleviate Drug-induced Hepatitis

**DOI:** 10.7150/thno.61214

**Published:** 2021-07-13

**Authors:** Qixiong Zhang, Shanshan Li, Lulu Cai, Yuxuan Zhu, Xingmei Duan, Peidu Jiang, Lei Zhong, Kun Guo, Rongsheng Tong

**Affiliations:** 1 Personalized Drug Therapy Key Laboratory of Sichuan Province, Department of Pharmacy, Sichuan Academy of Medical Science & Sichuan Provincial People's Hospital, School of Medicine, University of Electronic Science and Technology of China, Chengdu 610072, China; 2 College of Pharmacy, Southwest Minzu University, Chengdu 610000, China

**Keywords:** Self-assembly, Microenvironment-activatable, Injectable-nanoprodrug, Inflammation, Targeted therapy

## Abstract

Drug-induced hepatitis (DIH), which seriously interferes with disease treatment, is one of the most common reasons for termination of new drugs during preclinical studies or post-marketing surveillance. Although antioxidants and anti-inflammatory agents are promising, their nonspecific distribution and insolubility limit their application. Therefore, precise drug release at the disease site is an important way to alleviate DIH and avoid side effects.

**Methods:** A gripper-like hydrophilic cyclic phenylboronic acid (cPBA) was synthesized and a nanoprodrug (cPBA-BE) was established by coupling cPBA with hydrophobic baicalein (BE). The stimuli-responsive release properties and therapeutic effect of cPBA-BE on drug-injured hepatocyte were investigated. The biodistribution and therapeutic effect of cPBA-BE both in acetaminophen-induced acute hepatitis model and rifampicin-induced chronic hepatitis model were further evaluated.

**Results:** cPBA-BE conjugate could self-assemble into nanoprodrug with cPBA as the hydrophilic external layer and BE as the hydrophobic core. In HepaRG cells, cPBA-BE showed stronger cellular uptake. Due to the H_2_O_2_- and acid-sensitivity, cPBA-BE could achieve adequate BE release, significantly resist the depletion of GSH, mitochondrial dysfunction, downregulation of inflammation and cell apoptosis in the acetaminophen injured HepaRG cells. Biodistribution showed that cPBA-BE specifically increased the concentration of BE in the liver of DIH mice. cPBA-BE could alleviate acetaminophen-induced acute hepatitis or rifampicin-induced chronic hepatitis more effectively through relieving the oxidative stress, inflammation and block the neutrophil infiltration in liver.

**Conclusions:** cPBA is expected to be a good platform for constructing injectable nanoprodrug with both H_2_O_2_ and pH-responsive properties by coupling a wide range of drugs containing* o*-diol. In this study, the nanoprodrug cPBA-BE was determined to be effective for alleviating the DIH.

## Introduction

Drug-induced hepatitis (DIH) refers to the outbreak of liver inflammation caused by drugs or/and its metabolites 1. For healthy people with no history of hepatitis or other serious diseases, certain degree of liver damage may occur after taking some drugs 2-4. DIH is one of the most common reasons for termination of new drugs during preclinical studies and withdrawal of new drugs in post-marketing surveillance, resulting in serious clinical and financial problems 5. Therefore, DIH has become a severe public health problem that cannot be ignored, and presents unique challenges for patients, clinicians, and regulators. At present, there are more than 30,000 drugs and health products in our daily life, and more than 1,000 drugs can cause DIH 6. DIH can be further subdivided into acute and chronic hepatitis. For example, acetaminophen (APAP), one of the most widely used antipyretic and analgesic drug 7-9, could induce acute hepatitis by its toxic metabolite (N-acetyl-*p*-benzo-quinone imine, NAPQI) generated by hepatic cytochrome P450 (CYP450) system 10. Excessive NAPQI depletes glutathione (GSH), resulting in the covalent binding of cysteine, especially mitochondrial proteins 11. This could cause oxidative stress and dysfunction of mitochondria, then induce hepatic necrosis 12-15. Besides acute hepatitis, chronic hepatitis is also very common in clinical practice, especially in patients who require long-term medication 3. WHO recommends rifampicin (RFP) for the treatment of tuberculosis for at least 6 months 16. As a first-line anti-tuberculosis drug, RFP has been reported to cause chronic hepatitis in an endless stream which still remains a major global health problem 17. For instance, long‐term use of RFP could lead to liver injury with yellow gangrene, hepatomegaly and cholestasis 18, 19.

It has been well-recognized that oxidative stress, sterile inflammation and compensatory liver repair and regeneration were key signaling pathways during the development of DIH 20. Therefore, antioxidants and activators of antioxidation system have a significant alleviating effect on DIH 21. N-acetyl-*L*-cysteine (NAC) and GSH are recommended for all patients with DIH to decrease mortality 22. Besides, many other promising compounds have been explored in laboratory research. For example, shikonin could attenuate DIH via inhibition of oxidative stress and inflammation 23. Mangafodipir trisodium is endowed with superoxide dismutase, catalase, and glutathione reductase (GR) activities. It has also been reported that preventive or curative administration of mangafodipir trisodium to DIH mice significantly increases survival rates, and abrogates aspartate aminotransferase (AST) elevation and histological damage 24. *Scutellaria baicalensis Georgi* has been widely used as a medicinal plant in east Asian countries. Baicalein (BE) isolated from the root is considered to be an important activator of the nuclear factor-E2 related factor 2 (Nrf-2) pathway, which has been shown to play a key role in the treatment of DIH 25. Besides, BE could also relieve APAP-induced autophagy by regulating AKT/mTOR pathway, LC3B and p62 expression. The hepatoprotective effect of BE on APAP-induced liver injury was found to be involved in JAK2/STAT3 and MAPK signaling pathway 26. However, in the current studies, hydrophilic antioxidants or antioxidases are metabolized too fast to reach effective concentration 27, while hydrophobic therapeutic drugs, like BE, also have obvious disadvantages: 1) low bioavailability; 2) lack of organ specificity; 3) solvent side effects 28. These disadvantages have more or less limited their application in the treatment of DIH.

In recent years, nanosized drugs including micelles, polymer nanoparticles, liposomes, nanocrystals, etc. have been widely used in the prevention, diagnosis, and treatment of various diseases 29. Particularly, stimuli-responsive nanoparticles have attracted much attention for their potential applications in medicine because of their response to stimuli signals such as enzymes 30, glucose 31, GSH 32, pH 33 or reactive oxygen species (ROS) 34. Notably, due to the enhanced metabolic activity in inflammatory tissues, a microenvironment with low pH and increased ROS is a suitable trigger for targeting treatment of inflammatory diseases 35, 36. For the molecular structure of ROS-responsive materials, ROS-labile moieties mainly include propylene sulfide, thioether ketal, selenium- or tellurium-containing groups, peroxalates, oligoproline peptide, boronic acid esters, and Si-C covalent bonds 37. The molecular structure of pH-sensitive materials usually contains acid-base groups, such as carboxyl, amino, imine bonds, hydrazone bonds, etc 38.

**Scheme 1:**
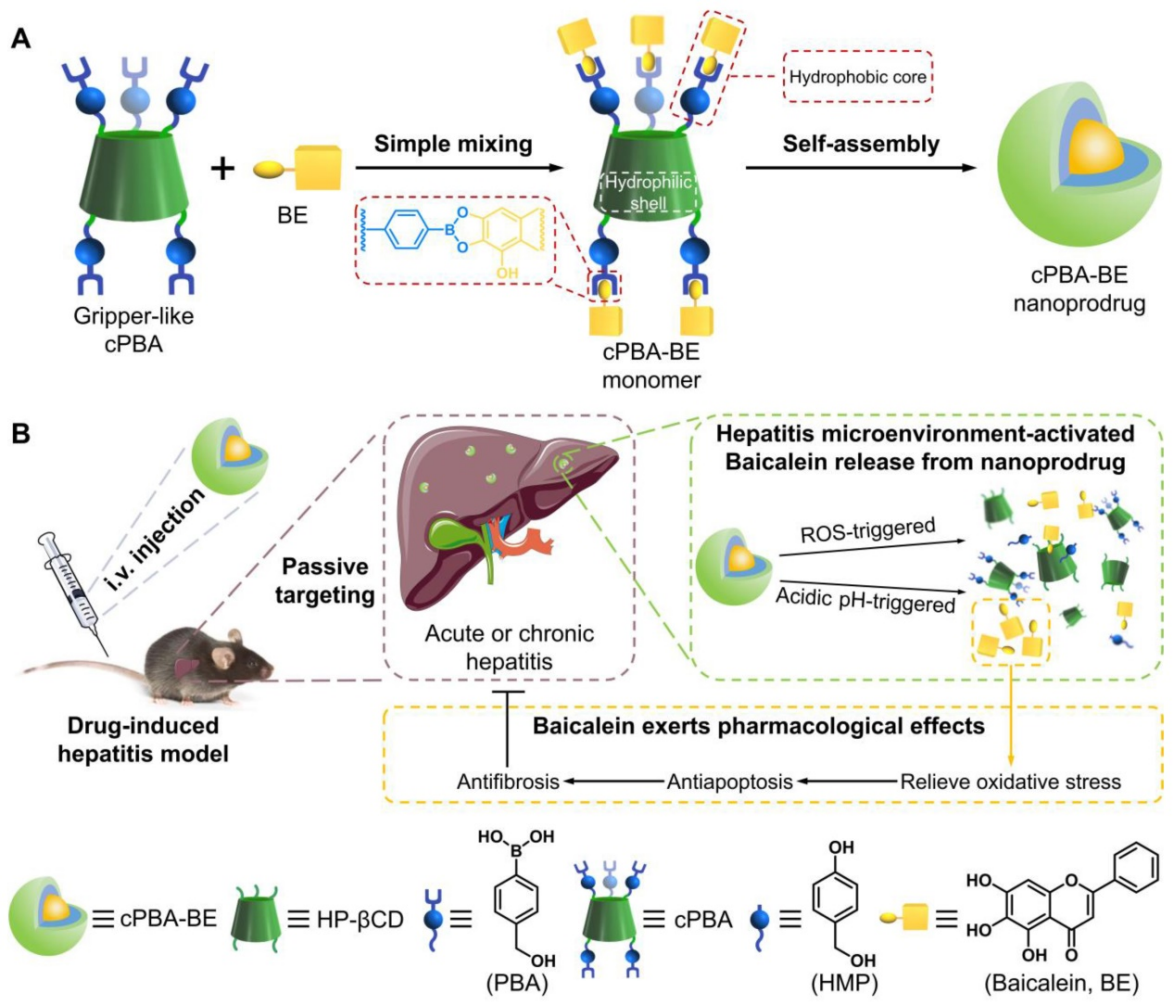
Schematic illustration for the preparation and application of the cPBA-BE nanoprodrug. Baicalein (BE) is coupled to the gripper-like circular PBA (cPBA) via the reaction between *o*-diol and PBA to form cPBA-BE amphiphilic monomer, then nanoprodrug cPBA-BE is formed by self-assembly (**A**). Passive and precise delivery of BE to the liver tissue of hepatitis. The nanoprodrug cPBA-BE is disassembled by excessive ROS and acidic environment in DIH to release the BE (**B**). HMP refers to 4-hydroxymethyl-phenol.

To integrate ROS-sensitive and pH-sensitive chemical moieties into one carrier material, phenylboronic acids (PBA) draws our attention. On the one hand, PBA is able to react with ROS resulting in the cleavage of the covalent bond between carbon and boron 39. On the other hand, PBA is a well-known coupling reagent for the compounds containing *o*-diol to form acid-sensitive boric acid ester bonds 40. Inspired by these features, a nanoprodrug that is both sensitive to ROS and acidic environment was designed (**Scheme 1**). Specifically, hydrophobic PBA was chemically bonded to water-soluble injection-grade hydroxypropyl-β-cyclodextrin (HP-βCD) to obtain a gripper-like hydrophilic cyclic PBA (cPBA). After the facile esterification between cPBA and BE, amphiphilic cPBA-BE monomer was spontaneously assembled into a nanoprodrug (cPBA-BE) in aqueous solution. Thus, cPBA-BE was expected to be cleaved both by ROS and acidic environment, then released BE at the inflammatory site. The biocompatibility of cPBA-BE has been preliminarily demonstrated in cell experiment and intravenous injection experiment in mice. In addition to evaluate the effect of cPBA-BE on drug-induced HepaRG cell injury, two kinds of mice models were established to demonstrate the therapeutic efficacy of cPBA-BE to acute and chronic DIH.

## Materials and Methods

### Regents and Materials

Injection grade hydroxypropyl-β-cyclodextrin (HP-βCD, *F_w_* = 1541.54, purity ≥ 98%) was purchased from Zhiyuan Biotechnology Co., Ltd. (China). Anhydrous N,N-dimethylformamide (DMF), anhydrous dichloromethane (DCM) and N,N'-carbonyldiimidazole (CDI) were obtained from J&K (China). 4-(Hydroxymethyl)phenylboronic acid (PBA) and 4-acetamidophenol (APAP) were supplied by Sigma-Aldrich (St. Louis, MO, USA). Rifampicin (RFP) was purchased from Tokyo Chemical Industry Co., Ltd. (Japan). Baicalein (BE) was purchased from MedChemExpress (purity > 98%). Cyanine5 NHS ester (Cy5) and Cyanine7.5 NHS ester (Cy7.5) were obtained from Lumiprobe (USA). William's E Medium, Dulbecco's Modified Eagle's Medium (DMEM), fetal bovine serum (FBS), penicillin, streptomycin and trypsin for cell culture were purchased from Gibco (USA). FITC Annexin V Apoptosis Detection Kits with 7-aminoactinomycin D (#640922) was purchased from Biolegend (USA), while MTT Cell Proliferation and Cytotoxicity Assay Kit (#C0009), Enhanced BCA Protein Assay Kit (#P0010S) and Reactive Oxygen Species Assay Kit based on DCFH-DA (#S0033) were supplied by Beyotime Biotechnology Co. Ltd. (China). N-(4-Diphenylphosphinophenyl)-N'-(3, 6, 9, 12-tetraoxatridecyl)perylene-3, 4, 9, 10-tetracarboxydiimide (LiperFluo™, #L248) was obtained by Dojindo Molecular Technologies (Japan). TMRE-Mitochondrial Membrane Potential Assay Kit (#ab113852) was obtained from Abcam (USA). Collagenase Ⅱ, DNAse I, Lymphoprep and D-Hank's were purchased from Solarbio (China). APC-labeled anti-mouse Ly6G antibody (#127613) and FITC-labeled anti-mouse CD11b antibody (#101205) were purchased from Biolegend (USA). With the exception of myeloperoxidase (MPO) ELISA kits (#E4580) was obtained from BioVision (USA), glutathione (GSH), glutathion reductases (GR), malondialdehyde (MDA), aspartate aminotransferase (AST), alanine aminotransferase (ALT) and lactate dehydrogenase (LDH) assay kit were purchased from Nanjing Jiancheng Bioengineering Institute (China). QuantiCyto^®^ Mouse ELISA kits for TNF-α and IL-1β were purchased from Neobioscience (China). Kits for H_2_O_2_ (#DIOX-250) were purchased from BioAssay Systems (USA). All the other reagents are commercially available and used as received.

### Synthesis and characterization of cPBA

According to previously established method with modification 39, the PBA-grafted HP-βCD material, namely, cyclic PBA (cPBA), was obtained through a two-step process. Briefly, PBA (199.0 mg, 1.31 mmol) and CDI (213.2 mg, 1.31 mmol) were dissolved in anhydrous DCM at room temperature (RT, 25 ℃). After constant magnetic stirring for 1 h, 20 mL DCM was added into the reaction mixture, followed by washing with 30 mL of deionized water for three times. The organic phase was further washed with saturated NaCl solution, dried over Na_2_SO_4_, and concentrated in vacuum to obtain CDI-activated PBA derivative (PBA-CDI). Subsequently, under the protection of N_2_, HP-βCD (725 mg, 0.47 mmol), TEA (250 μL, 1.8 mmol), and PBA-CDI (347 mg, 1.41 mmol) were dissolved in anhydrous DMF. The mixture was magnetically stirred overnight at RT. The final product cPBA was precipitated from cooled ether, collected by centrifugation at 5,500 *g* for 5 min, and dried to give a white powder. Then, 30 mg cPBA were dissolved in D_2_O in tubes for ^1^H NMR analysis. Fourier-transform infrared (FT-IR) spectroscopy of cPBA and HP-βCD were performed on a PerkinElmer spectrometer (100S, USA). The above characterization tests were performed at room temperature (25 ℃).

### Preparation and characterization of nanoprodrug cPBA-BE

cPBA-coupled baicalein nanoprodrug (cPBA-BE) was formulated by self-assembly 41. Briefly, cPBA (200 mg, 0.076 mmol) was added into 4 mL anhydrous DMSO containing BE (145 mg, 0.54 mmol). After stirring overnight with molecular sieves, the solution was injected into 16 mL dulbecco's phosphate buffered saline (DPBS, pH 7.4) under ultrasound to promote self-assembly. Prior to subsequent use, DMSO was removed by dialysis using dialysis bag (MWCO = 1,000 Da) against 1 L DPBS for 48 h, and replaced with fresh DPBS every 4 h. The final solid nanoprodrug cPBA-BE was obtained by lyophilization. Similarly, Cy5- or Cy7.5-loaded cPBA-BE solution can be obtained by adding hydrophobic cyanine dyes in the self-assembly process.

Dynamic light scattering (DLS) and ζ-potential measurements were performed on a Malvern Zetasizer Nano ZS Instrument at 25 ℃. Morphological study was performed by transmission electron microscopy (TEM).

To measure the drug loading content of nanoprodrug, 200 mg lyophilized cPBA-BE was complete hydrolyzed in acetonitrile with 1 mM H_2_O_2_ at pH 6.0. The concentration of BE in hydrolysate was quantified by LC-MS referring to the standard curve 42. The detection wavelength of BE is 272 nm, and the target molecular ion peak [M+H]^+^ is 271.06 m/z. The mobile phase was 75% acetonitrile and the flow rate was 1 mL/min. Similarly, cPBA-BE-Cy5 and cPBA-BE-Cy7.5 were hydrolyzed completely in acetonitrile with 1 mM H_2_O_2_ at pH 6.0, the concentrations of dyes were determined by UV-Vis spectrophotometry at 646 and 788 nm, respectively.

Calculate the drug loading content (DLC) according to the following formula:

DLC = 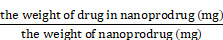 × 100%

### Characterization of self-assembly and coupling between cPBA and BE

According to the previously reported method 43, the intensity of scattered light will increase suddenly when the nano-assembly appears. The critical aggregation concentration (CAC) of BE was determined by DLS in DPBS. A series of solutions of BE ranging from 0.003 to 35 μg/mL were prepared from a DMSO stock solution of BE. The CAC was estimated as the cross-point when extrapolating the intensity in the low and high concentration ranges. In this assay system, the concentration of cPBA is 2 mg/mL.

A binding profile between cPBA and BE was measured by monitoring the quenching of intrinsic fluorescence of PBA moiety. Briefly, 2 mg/mL cPBA containing 0, 3, 6, 12 or 24 μg/mL BE were prepared in DMSO (pH 7.4). The fluorescence intensity of PBA moiety (*Ex* = 302 nm, *Em* = 388 nm) was measured and plotted with Stern-Volmer equation 41. In another parallel experiment, the pH of the DMSO solvent was adjusted to 5.6 using HCl and then the fluorescence of PBA was detected by the same procedure.

### Stability and hydrolysis of cPBA-BE* in vitro*

The stability of cPBA-BE nanoprodrug against salt concentrations (0-600 mM NaCl) or serum (10% FBS) was investigated from the perspectives of hydrodynamic diameter and polydispersity by DLS. Additionally, since phenylboronic acid structure is sensitive to ROS or pH 44, the morphology of cPBA-BE in the presence or absence of H_2_O_2_ in PBS with different pH values (pH 5.6 to 11.0) were observed by TEM.

### Drug release profiles of cPBA-BE in various media

To examine the sensitivity of nanoprodrug cPBA-BE to ROS, various ROS generators were prepared as described previously 45. Aqueous solutions containing either H_2_O_2_ or OCl^-^ were prepared based on commercial agents. Hydroxyl radical (•OH) was generated by the Fenton reaction between ferrous acetate and H_2_O_2_, while superoxide anion (O_2_^-^) was produced by the xanthine-xanthine oxidase system. Peroxynitrite (ONOO^-^) was obtained by the reaction of H_2_O_2_ and nitrite. cPBA-BE at 10 mg/mL was separately incubated in 5 mL medium with or without different ROS for 12 h. The released BE was detected by LC-MS.

The hydrolysis behavior of cPBA-BE (10.0 mg/mL) was examined in 5 mL PBS (10 mM, pH 7.4) containing various concentrations (0, 0.125, 0.25, 0.5, 1 mM) of H_2_O_2_ at 37 °C. Quantitative experiments were conducted by measuring the absorbance of cPBA-BE containing aqueous solutions at 500 nm after incubation for different time (0, 5, 10, 15, 20, 25, 30, 40, 60, 80, 120 and 150 min). Similarly, hydrolysis of cPBA-BE in buffer solutions with different pH (varying from pH 5.6 to 11.0) was studied. Meanwhile, the release of BE from cPBA-BE was also investigated in 0.9% saline containing 6 mmol/L glucose which simulating normal human blood glucose level, or high salt concentration solution (0.6 mol/L NaCl).

### Cell culture

Human hepatic stem cell line HepaRG is the most suitable cell line for studying drug-induced hepatitis because of the richer CYP450 proteins in HepaRG than L-02, HepG2 and hiHeps cell lines 46, 47. HepaRG was obtained from ThermoFisher Scientific (#HPRGC10). The cells were seeded at 1 × 10^5^ undifferentiated cells/cm^2^ in Hepatocyte Wash Medium (ThermoFisher Scientific, #17704024) containing additives for growth (Gibco). Then the cells were cultured at 37 °C with 21% O_2_ and 5% CO_2_ for 14 days before differentiation. The medium was renewed every 3 days. Cell differentiation was induced as described 48. Briefly, the growth medium was composed of William's E medium supplemented with 10% FBS, 100 units/mL penicillin, 100 μg/mL streptomycin, 5 μg/mL insulin, and 50 μM hydrocortisone hemisuccinate. For the routine differentiation process, a two-step procedure was used. Cells were first maintained in the growth medium for 2 weeks, then maintained in the differentiation medium (supplemented with 2% DMSO) for 2 more weeks. The cells were maintained up to 4 weeks after differentiation for use. As for the mouse macrophage RAW264.7 cells, they were cultured in DMEM-based complete medium.

### Cytotoxicity evaluation

HepaRG or RAW264.7 cells were planted at 1 × 10^4^ cells/well in 96-well plates for 24 h before sample was added. Subsequently, cells were treated with the medium containing BE, cPBA and cPBA-BE respectively at various concentrations (7.8, 15.6, 31.25, 62.5, 125, 250, 500, 1000 μg/mL) for 12 h. The cell viability was quantified by the methyl thiazolyl tetrazolium (MTT) assay.

### Intracellular uptake and drug release profile of cPBA-BE in HepaRG cells

HepaRG cells were seeded in 12-well plates at a density of 4 × 10^5^ cells/well in 1 mL of complete medium. After 24 h, the medium was replaced with 1 mL of fresh medium containing 10 μg/mL Cy5-loaded cPBA-BE (cPBA-BE-Cy5) and incubated for various periods of time (0.5, 1, 2, 4, 6, 12 and 24 h). Then, the cells were digested and fluorescence intensity was determined using a flow cytometer (BD Accuri C6). Through similar procedures, dose-dependent (1.25, 2.5, 5 and 10 μg/mL cPBA-BE-Cy5) internalization profiles were examined after 2 h of incubation.

Meanwhile, the drug release of cPBA-BE in cells was examined by a parallel test. 80 μg/mL cPBA-BE was incubated with HepaRG cells for 8 h. Then replaced with fresh medium, the cells were digested and menthol/acetonitrile (1:1) was added to extract the drug. The content of BE was analyzed by LC-MS with 75% acetonitrile as mobile phase and the flow rate was 1 mL/min.

To further verify the drug release of cPBA-BE in response to oxidative stress, HepaRG cells were incubated with 80 μg/mL cPBA-BE in the presence or absence of 3 mg/mL APAP for 8 h. Thereafter, the intracellular drug concentration was determined by the same method.

### Biological activity of cPBA-BE in HepaRG cells

#### Anti-oxidative and anti-inflammatory activities

HepaRG cells were cultured in 12-well plates at a density of 4 × 10^5^ cells/well with William's E Medium-based complete medium for 12 h. Then the medium was changed and cells were pre-cultured with 3 mg/mL APAP for 4 h. After which, the medium was placed with 80 μg/mL cPBA-BE or 20 μg/mL BE (according to the drug loading content: 25.5% w/w) for another 4 h. PBS-treatment was used as control group. Subsequently, cells were washed three times with Hank's balanced salt solution (HBSS) and treated with 10 μM DCFH-DA in the dark at 37 °C for 40 min. After removing HBSS, quantitative analyses were conducted by flow cytometry. 20 μM LiperFluo^™^ was used as a probe for lipid peroxides (LPO) with the similar operation steps. Tetramethylrhodamine ethyl ester (TMRE) was used to analyze mitochondrial membrane potential (ΔΨm). Specifically, HepaRG cells were seeded into 6-well plates and pre-incubated with 3 mg/mL APAP for 4 h, then treated with 80 μg/mL cPBA-BE or 20 μg/mL BE for another 4 h. After which, cells were trypsinized, washed, and incubated with 100 nM of TMRE in PBS at 37 °C for 30 min. After washing twice by PBS, the cells were assayed by flow cytometry.

In a parallel experiment, after incubation with each sample, the cell lysate was centrifuged and supernatant was collected. The GSH and GR, as oxidative stress indicators, were measured using commercial kits. A typical proinflammatory factor TNF-α was determined by ELISA.

#### Detection of damage biomarkers

Cell death can be assessed by lactate dehydrogenase (LDH) release according to kit instructions and report 49. Especially for HepaRG cells, LDH release is a more sensitive parameter of cell damage because of the low level of alanine aminotransferase (ALT) activity. Another recognized important damage parameter is aspartate aminotransferase (AST). The LDH level and AST level were estimated using LDH assay kit and AST assay kit, respectively according to the manufacturer's instructions. The relative comparison was performed using the LDH level and AST level in blank control group as standards.

#### Anti-apoptotic activity

Apoptosis analysis was conducted using FITC Annexin V Apoptosis Detection Kit with 7AAD according to the manufacture's protocol. Pre-incubation with 3 mg/mL APAP for 4 h resulted in significant apoptosis of HepaRG cells. Then the medium was changed to 80 μg/mL cPBA-BE or 20 μg/mL BE for another 4 h. The protective effect of cPBA was investigated by MTT method. After HepaRG cells were treated with 3 mg/mL APAP for 4 h, the culture medium was replaced with cPBA of different concentrations (6.25, 12.5, 25, 50, 100, 200 μg/mL) for another 4 h. Then the cell viability was measured.

### Animals

All animal experiments were performed in accordance with the Guide for the Care and Use of Laboratory Animals proposed by the National Institutes of Health. All procedures and protocols were approved by the Animal Ethics Committee at Sichuan Provincial People's Hospital. Male C57BL/6 mice (18-20 g) and male Sprague-Dawley rats (200 g) were obtained from Sichuan Provincial People's Hospital. Animals were housed in standard mouse cages under conditions of optimum light, temperature, and humidity, with *ad libitum* access to water and food. Before further experiments were performed, all mice were acclimatized for at least 7 days.

### Hemolysis and toxicity evaluation of cPBA-BE in mice

Hemolysis induced by each sample was evaluated by measuring the release of hemoglobin from the erythrocytes. Briefly, fresh blood from Sprague-Dawley rats was diluted 10 folds in saline, and subsequently centrifuged at 2,000 rpm for 15 min to collect erythrocytes. 5 mg/mL free BE in 5% DMSO, 20 mg/mL cPBA in saline and 20 mg/mL cPBA-BE in saline were added in the blood cells, respectively. The resulting suspensions were incubated at 37 ℃ for 6 h, and then centrifuged at 2,000 rpm for 15 min. The absorbance of supernatants was measured at 540 nm in order to calculate the hemoglobin release. dd water was utilized as positive control (100% hemolysis).

Moreover, after injection of 100 μL cPBA-BE solution (20 mg/mL) via tail vein daily for 12 consecutive days, the blood sample and major organs were collected. The collected organs were fixed in 4% (v/v) buffered formalin for 1 day and embedded in paraffin. Tissue slides of 5 μm thick sections were prepared and stained with hematoxylin and eosin (H&E). Histology was evaluated by microscope. The collected blood samples were analyzed by hematological analysis (Sysmex KX-21, Sysmex Co., Japan). Biomarker molecules relevant to liver functions (AST and ALT) and kidney functions (creatinine, CREA; blood urea nitrogen, BUN) were quantified.

### Mice models of drug-induced acute hepatitis and chronic hepatitis

The mice model of acute hepatitis was induced by 4-acetaminophen (APAP). The APAP solution was prepared by dissolving APAP in sterilized saline at 20 mg/mL 50. Male C57BL/6 mice were fasted for 15 h before experimentation. Then, the mice received a single *i.p.* injection of 200 mg/kg APAP and were randomly divided into 3 groups (n = 5). After 6 h of APAP treatment, mice received *i.v.* injections of 100 μL various samples: PBS, free BE dissolved in 5% DMSO in saline v/v (5.1 mg/mL) and cPBA-BE dissolved in PBS (20 mg/mL). Healthy mice without APAP treatment were set as control group.

In addition, the mice model of chronic hepatitis was induced by rifampicin (RFP). The RFP solution was prepared by suspending it in 0.5% sodium carboxymethyl cellulose. Male C57BL/6 mice were fasted for 1 day before experimentation and randomly divided into 3 groups (n = 5). Mice were once daily *i.g.* administrated with 200 mg/kg RFP suspension for 12 consecutive days 51. Each day after administrating RFP for 4 h, mice received *i.v.* injections of 100 μL different samples: PBS, free BE dissolved in 5% DMSO in saline v/v (5.1 mg/mL) and cPBA-BE dissolved in PBS (20 mg/mL). Healthy mice without RFP treatment were set as control group.

### Targeting and pharmacokinetic study of cPBA-BE in APAP-induced hepatitis (AIH) mice

*Ex vivo* fluorescence imaging experiments were carried out to verify the targeting properties of cPBA-BE. After AIH model was established for 6 h, PBS, Cy7.5, and cPBA-BE-Cy7.5 (calculated as 0.5 mg Cy7.5) were intravenously injected, respectively. Then after 24 h, the mice were sacrificed to collect the liver, which was imaged immediately using an IVIS Spectrum living imaging system (*E*_x_/*E*_m_, 745/820 nm; exposure time, 2 s; binning, 8; F/Stop, 1). Fluorescence intensity of the liver tissue was analyzed by Living Image Software. Healthy mice were intravenously injected with cPBA-BE-Cy7.5 as a control group.

The concentration-time relationships of BE in liver and blood were focused in the pharmacokinetic studies. AIH mice were injected with 100 μL 5.1 mg/mL BE or 20 mg/mL cPBA-BE (equal to 5.1 mg/mL BE) through tail vein. Blood samples and liver tissues were collected at predefined time points (0.25, 0.5, 0.75, 1, 1.25, 1.5, 1.75, 2, 3, 4, 6, 8, 12, 16 and 24 h). According to previously reported methods with modification 43, the whole blood samples were immediately centrifuged at 12,000 *g* for 2 min to obtain plasma. Then 800 μL of ice-cold methanol was added into 200 μL of plasma to precipitate protein. After centrifugation at 12,000 *g* for another 2 min, the supernatant was purged with nitrogen, reconstituted with methanol, and detected by LC-MS. The mobile phase was consisted of acetonitrile and water at 75:25 (v/v). To determine the concentration of BE in liver, the liver tissues were homogenized and centrifuged. BE in the supernatant was extracted with methanol/acetonitrile then quantified by LC-MS. Typical pharmacokinetic parameters such as the maximum concentration (*C*_max_) in plasma or liver, time to reach *C*_max_ (*T*_max_), and the area under the plasma/liver BE concentration-time curve (*AUC*) were calculated.

### Evaluation of the therapeutic efficacy of cPBA-BE on AIH

#### Quantification of oxidative and inflammatory mediators in liver tissues

After administrated with different samples for 18 h, mice were euthanized and livers were isolated and weighed. The ratio of liver tissue to mouse body weight was calculated as the liver index. Additionally, liver tissues were homogenized in cold PBS with a tissue-tearor homogenizer and then centrifuged at 16,000 *g* for 10 min at 4 °C. The resulting supernatants were collected for quantification of the levels of oxidative mediators (H_2_O_2_, GSH, MDA, MPO) by commercial kits and inflammatory mediators (TNF-α and IL-1β) by ELISA. The total protein was measured by BCA kit.

#### Quantification of neutrophils in liver tissues

Hepatic leukocytes were purified as described 52, 53. In briefly, livers were excised and finely minced in digestive media containing 0.05% collagenase and 0.002% DNase I in D-Hank's. After gentle agitation at 37 °C for 30 min, the concentrate was passed through a 30-μm nylon mesh (Corning) and washed twice with ice-cold PBS (pH 7.4) and centrifuged at 300 *g* for 10 min. Lymphocytes were purified by layering the cell suspension on Lymphoprep^™^ and centrifuged at 800 *g* for 20 min at room temperature. The emigrated neutrophils in livers were analyzed by flow cytometry. Specifically, the suspension concentration was adjusted to 1 × 10^6^ cells/mL and labeled for 30 min with the following antibodies: APC-conjugated anti-mouse Ly6G antibody and FITC-conjugated anti-mouse CD11b antibody. The cells were collected in a flow cytometry (Accuri C6, BD) and analyzed using FlowJo 10 software.

#### Serum biomarker molecules of liver and kidney functions

After administrated with different samples for 18 h, mice were euthanized and the blood was obtained from the orbits of mice for hematological analysis and quantification of biomarker molecules relevant to liver functions (AST, ALT, LDH and ammonia) and kidney functions (CREA, BUN).

#### Histological assessment

The livers were fixed in 4% (v/v) buffered formalin for 1 day and embedded in paraffin. Tissue slides of 5 μm thick sections were prepared and stained with H&E for microscopic examination. Each slide was evaluated by an investigator blinded to the experimental groups. The hepatitis was scored according to the method previously described 54. Briefly, hepatitis was scored for the degree of edema (0-4), inflammatory infiltration (0-6), hemorrhage (0-6), and ballooning (pre-necrotic degeneration; 0-8). The total area of edema, infiltration, hemorrhage, and ballooning was also taken into account. The summation of the individual histological parameters resulted in a single score per slide with a maximum score of 24. For immunohistochemical assessment, sections were subjected to Masson trichrome (MT) staining 55.

#### Apoptosis assessment

The collected liver tissues were fixed in optimal cutting temperature compound (O.C.T) immediately, and 7-μm cryosections were made. Apoptotic hepatocytes were detected by terminal deoxynucleotidyl transferase-mediated deoxyuridine triphosphate nick end labeling (TUNEL) assay using the in situ cell death detection kit (Roche Diagnostics, USA). Images were acquired using an Olympus fluorescence microscope equipped with a Hamamatsu ORCA03G digital camera.

### Evaluation of the efficacy of cPBA-BE on RFP-induced hepatitis (RIH)

According to the aforementioned protocol with modification 51, a model of chronic drug-induced hepatitis caused by RFP was established. Evaluation of the efficacy of cPBA-BE on this model was mainly focused on liver organ index, histopathological score, and LPO level in liver tissue. In addition, the serum AST, ALT and LDH indicators were detected to evaluate the degree of RIH. Each measurement method is the same as that of AIH model.

### Statistical Analysis

Data are expressed as mean ± standard deviation (SD). Statistical analysis was assessed using one-way ANOVA test. A value of p < 0.05 was considered statistically significant.

## Results and Discussion

### Synthesis and characterization of cPBA based on HP-βCD

HP-βCD is a FDA-approved injectable hydrophilic derivative of βCD, a cyclic oligosaccharide with good *in vitro* and *in vivo* safety profiles 56. βCD and their derivatives have already been widely used in different drug formulations due to the unique hydrophobic cavity of cyclodextrins, which could form inclusions with insoluble drugs to improve its water solubility 57. However, simple inclusion complexes cannot achieve targeted and on-demand drug release. According to our previous reports 39, 45, 58, 59, a stoichiometric amount of H_2_O_2_ may be consumed with hydrolysis of phenylboronic acid derivatives (PBAs). PBAs were utilized as H_2_O_2_-responsive moieties to synthesize ROS-responsive anti-inflammatory materials. Moreover, the ester bonds formed by PBAs and *o*-diol are easily broken in acidic environments, which is often used to prepare pH-responsive materials 60-62.

**Figure 1:**
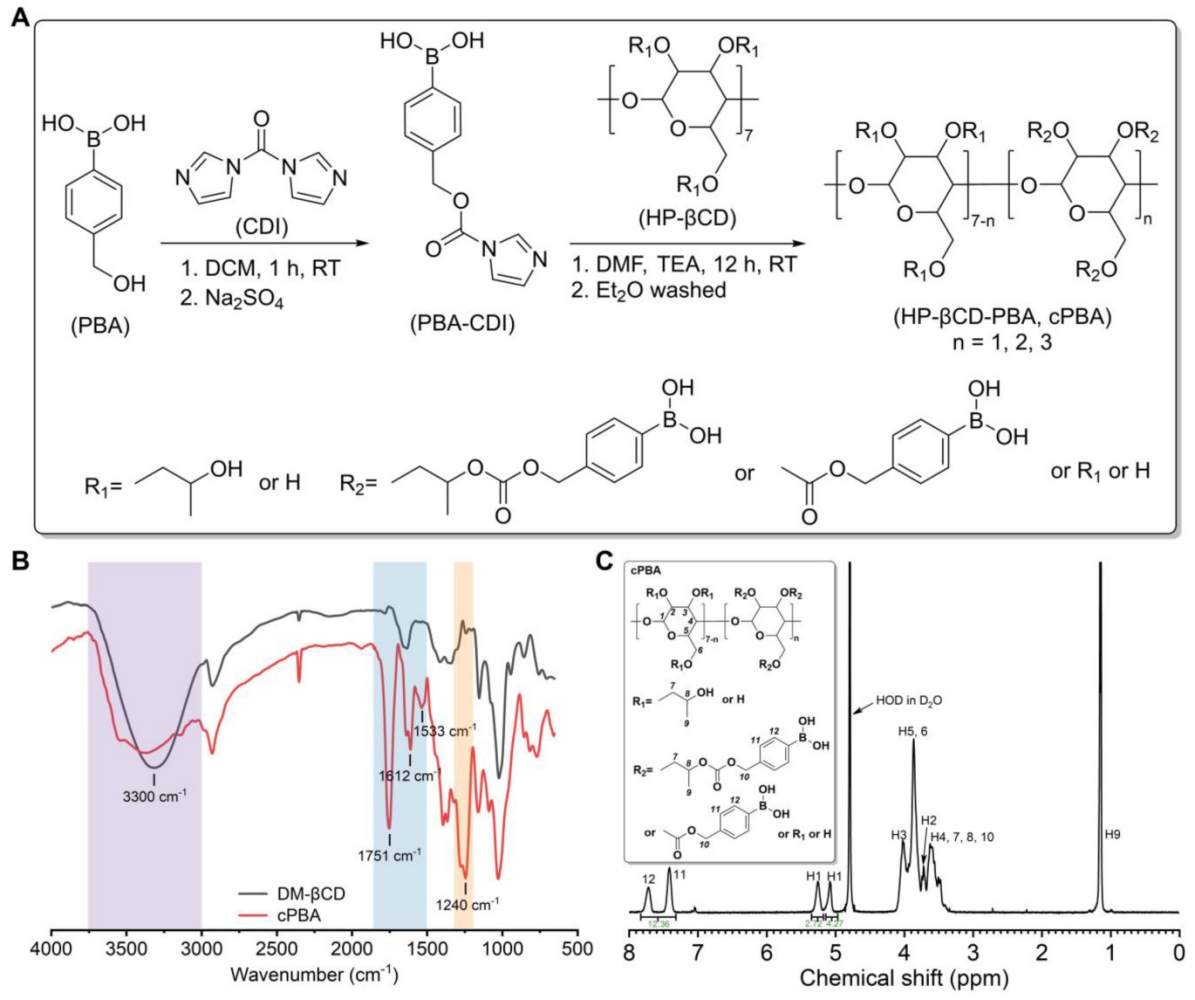
Synthesis and characterization of cPBA. **A**, The synthetic route of cPBA. **B-C**, The FT-IR (B) and (C) ^1^H NMR spectra in D_2_O of cPBA.

To establish the dual microenvironment-activatable prodrug, 4-(hydroxymethyl)phenylboronic acid (PBA) was grafted onto HP-βCD to form cyclic PBA (cPBA). The synthetic route of cPBA based on HP-βCD was shown in **Figure 1A**.

CDI was primarily reacted with PBA to form an active intermediate (PBA-CDI), then the imidazole group of PBA-CDI was subsequently substituted by hydroxyls of HP-βCD to form cPBA (yield 78.2 %). In the FT-IR spectrum of cPBA (**Figure 1B**), the severely attenuated absorption at 3300 cm^-1^ indicated that 3-hydroxyl groups were substituted. The typical signals relevant to carbonyl (at 1751 cm^-1^) and phenyl groups (at 1612 cm^-1^ and 1533 cm^-1^) were obviously observed. Additionally, a clear absorption at 1240 cm^-1^ represents a newly formed carbonate bond. This was further confirmed by the ^1^H NMR spectrum (**Figure 1C**), in which signals from PBA could be clearly observed. Calculation based on the ^1^H NMR spectrum revealed that about three PBA moieties were linked to each HP-βCD.

It is worth noting that due to the extremely hydrophilic properties of HP-βCD (*S_HP-βCD_* > 50 g/100 g H_2_O) and the limited grafting number of PBA moieties, cPBA could be dissolved in polar organic solvents such as DMSO and methanol, even in water (*S_cPBA_* > 10 g/100 g H_2_O). Above all, the water-soluble cPBA was successfully and facilely synthesized.

### Coupling between cPBA and BE

**Figure 2:**
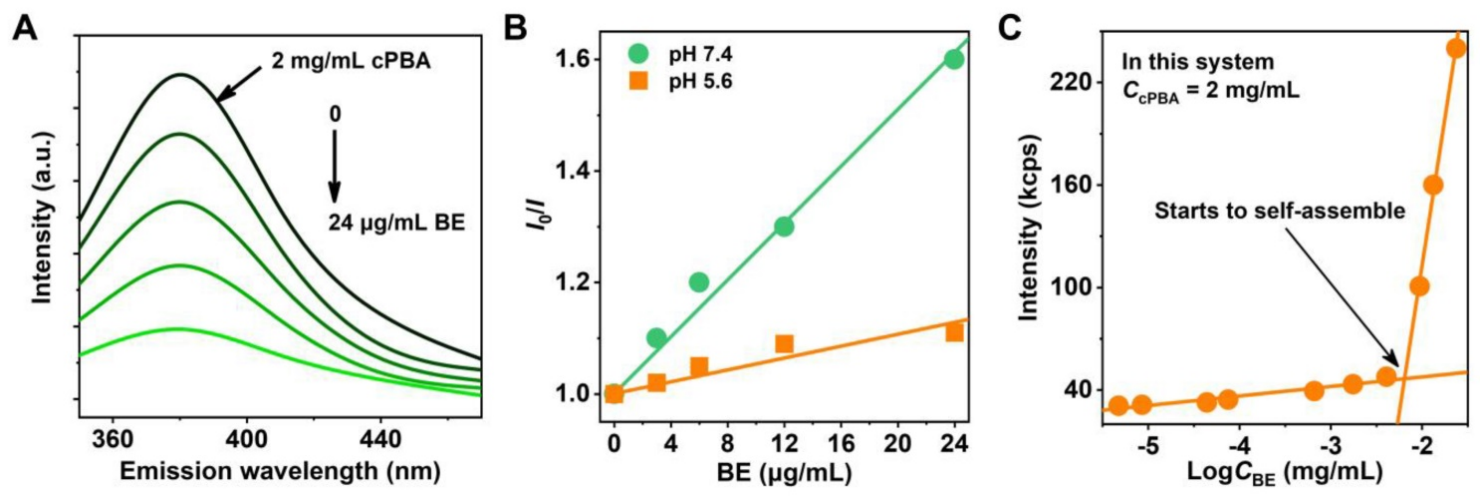
Interaction between cPBA and BE. **A**, Fluorescence quenching of cPBA after an interaction with various concentrations (0, 3, 6, 12, 24 μg/mL) of BE. **B**, Plotting of BE concentration-dependent steady-state fluorescence quenching of cPBA using the Stern-Volmer equation at different pH. *I*_0_ and *I* represent the fluorescence intensity without or with different concentrations of BE, respectively. **C**, Detecting the lowest concentration of BE for cPBA-BE self-assembly by dynamic light scattering. 2 mg/mL cPBA was in the system.

Theoretically, BE with *o*-diol moiety could react with hydrophilic cPBA through boric ester bond to improve its hydrophobic properties. It is known that after interacted with *o*-diol, the intrinsic fluorescence of PBA would be quenched 41. The cPBA emits fluorescence at a wavelength of 388 nm when excited by light at 302 nm. As shown in **Figure 2A**, a steady-state quenching of cPBA emission fluorescence was observed with the increase of BE. When 24 μg/mL BE was present in the system, the fluorescence intensity was only 20.5 % of that in 2 mg/mL cPBA. Meanwhile, a pH-dependent quenching was measured and plotted using the Stern-Volmer equation 63. The ratio of the fluorescence intensity of cPBA without BE (*I*_0_) to that of cPBA with different concentrations of BE (*I*) was shown in **Figure 2B**. Compared with pH 7.4, a lower slope at pH 5.4 represents that acidic condition is unfavorable for the coupling between cPBA and BE. This is consistent with the binding characteristics between boric acid and diol.

For nano-drug delivery systems, it is critical to keep formulated nanoprodrug with high stability and sensitivity, even at very low concentration under intense dilution of the physiological fluids. The mutation of intensity of scattered light at 500 nm is an important signal for the formation of nanoaggregates. At low concentrations of BE, the detected scattering intensity of the system (containing 2 mg/mL cPBA) is approximately equal to a low constant value. At higher concentrations of BE, the intensity increased linearly with the increasing concentration of BE. The intersection of the best fit lines drawn by the data represents the mutation point occurred at about 6.5 μg/mL BE (**Figure 2C**). The relatively low concentration of BE indicates that it is very easy to form stable cPBA-BE nanoprodrug in aqueous solution. This may due to the three phenolic hydroxyls of BE provided double reaction opportunities (two *o*-diols), which is beneficial to improve the coupling efficiency between cPBA and BE.

### Preparation and characterization of nanoprodrug cPBA-BE

**Figure 3:**
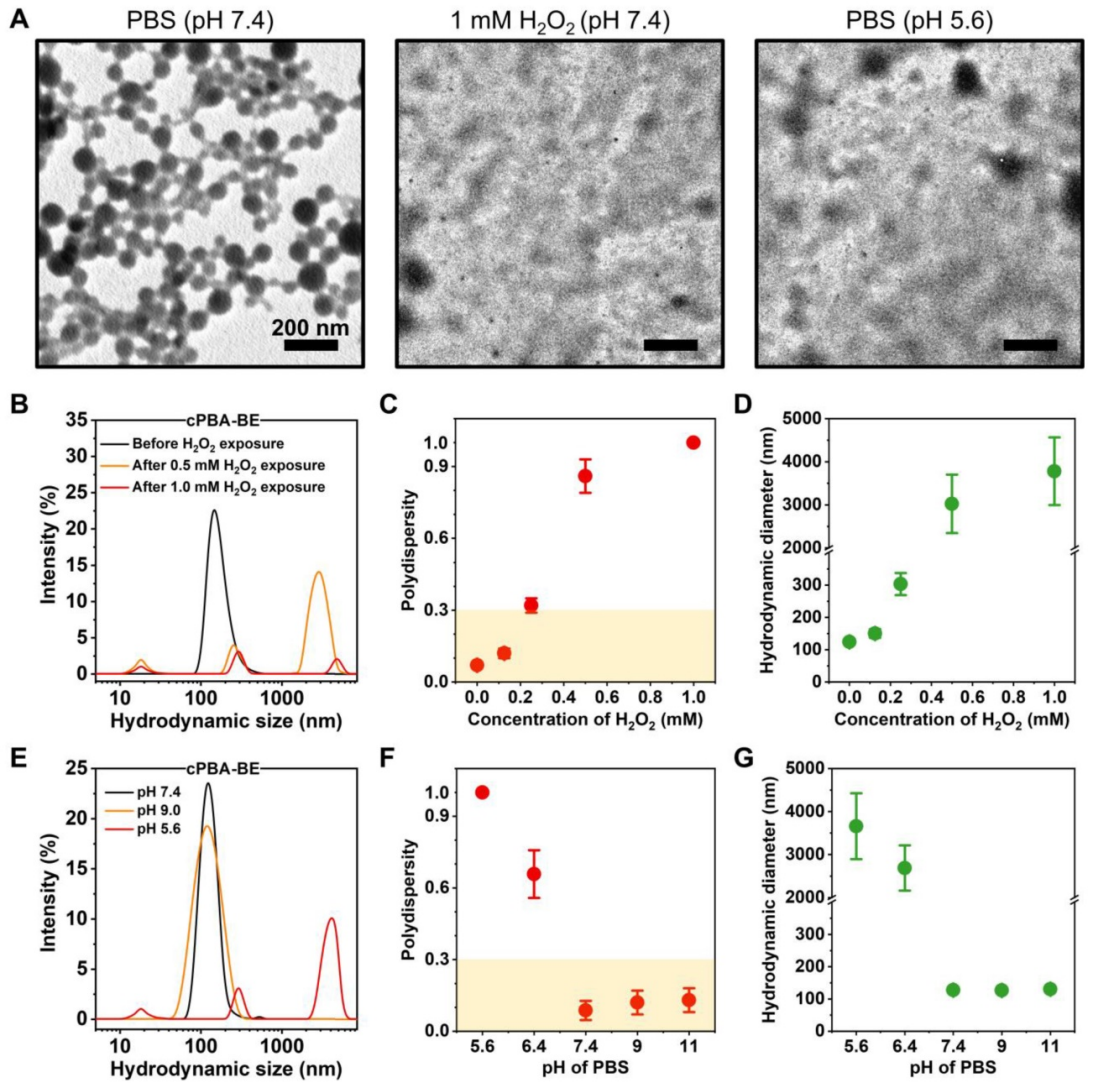
Characterization of cPBA-BE nanoprodrug. **A**, TEM images of cPBA-BE in different media. **B-D**, Size distribution (B), polydispersity (C) and average hydrodynamic diameter (D) of cPBA-BE exposed in various concentrations (0, 0.125, 0.25, 0.5 or 1 mM) of H_2_O_2_. **E-G**, Size distribution (E), polydispersity (F) and average hydrodynamic diameter (G) of cPBA-BE in PBS with various pH (5.6, 6.4, 7.4, 9.0 or 11.0). All data are presented as mean ± SD (n = 6).

cPBA-BE complex was expected to be amphiphilic, which may be able to self-assemble into nanoparticles in aqueous media without adding any surfactants. Characterization of TEM suggested that the as-prepared cPBA-BE nanoprodrug exhibited a well-defined uniform spherical shape in pH 7.4 PBS. However, after incubated with PBS at pH 5.6 or containing 1 mM H_2_O_2_, the spherical shape of cPBA-BE nanoprodrug disappeared and transformed to crystals with different sizes (**Figure 3A**).

Hydrodynamic sizes of cPBA-BE measured by DLS in different media were shown in **Figure 3**. In PBS at pH 7.4 without H_2_O_2_, cPBA-BE exhibited narrow particle size distribution around 100 nm. However, after cPBA-BE exposed to different concentrations of H_2_O_2_, multiple particle size ranges appeared. Especially with 1 mM H_2_O_2_, the intensity of scattered light decreased dramatically (**Figure 3B**). Polydispersity (PDI) as an important indicator of particle size uniformity, less than 0.3 is considered to be monodispersed 64. The PDI reached 0.32 ± 0.03 when 0.25 mM H_2_O_2_ was present in the PBS buffer. With further increase of H_2_O_2_, the “chaos” degree of the solution continued to break out. The specific PDI (>1.0) couldn't even be measured when the concentration of H_2_O_2_ greater than 1 mM (**Figure 3C**). Meanwhile, exposure to 0.25 mM H_2_O_2_ dramatically increased the average hydrodynamic diameter of cPBA-BE from 124.2 ± 2.2 nm to 303.2 ± 34.2 nm. Further increase of the H_2_O_2_ concentration results larger hydrodynamic diameters (**Figure 3D**). These results revealed the H_2_O_2_-responsive disassembly behavior of cPBA-BE nanoprodrug. Uniform particle size (around 100 nm) of cPBA-BE was presented at pH 7.4 and pH 9.0 (**Figure 3E**). Alkaline medium (pH higher than 7.4) didn't significantly influence the monodispersity and hydrodynamic particle size of cPBA-BE (**Figure 3F-G**). In contrast, in an acidic environment (pH 5.6), cPBA-BE exhibited three particle size distribution ranges, with significantly increased PDI and hydrodynamics diameter (**Figure 3F-G**). Consistently, the results reflected the acid-activated disassembly ability of cPBA-BE.

For nanoscale assemblies, the concentration of ions in physiological fluids may also affect its stability. Thus, the stability of cPBA-BE under different concentrations of NaCl was determined by DLS. Though the size and PDI of cPBA-BE slightly increased with the increasing NaCl concentrations, the average size mainly ranges from 125 to 133 nm and the PDI was less than 0.3 (**Figure S1A-B**). It indicated that cPBA-BE nanoprodrug is stable in NaCl solutions. Moreover, incubated in the medium with 10% FBS shows no significant change in the hydrodynamic diameter of cPBA-BE (**Figure S1C**). Therefore, even with the high concentration (600 mM) of NaCl or 10 % FBS, the nanostructure of cPBA-BE would not be destructed.

### ROS- and pH-activated drug release profiles of cPBA-BE

**Figure 4:**
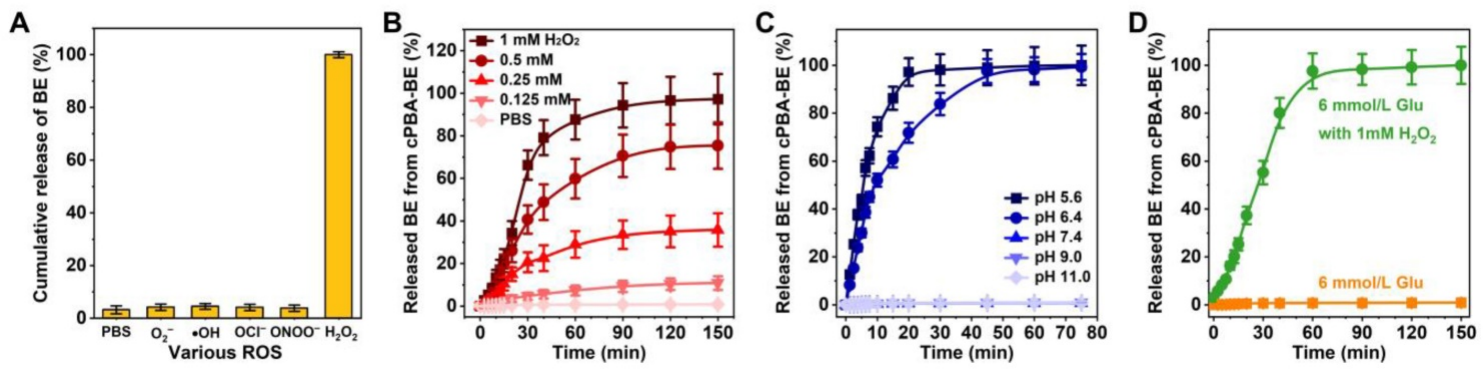
Microenvironment activated drug release. **A**, Sensitivity of cPBA-BE against various ROS. **B-C**, BE release profiles in PBS with various concentrations of H_2_O_2_ (B) or at different pH values (C). **D**, Effect of glucose on BE release from cPBA-BE. Normal blood glucose concentration is about 6 mmol/L. All data are presented as mean ± SD (n = 6).

The dual-responsive hydrolysis properties of cPBA-BE suggested its ability to release BE under certain environment. The contents of BE released from cPBA-BE nanoprodrug in PBS without or with five kinds of ROS (O_2_^-^, •OH, OCl^-^, ONOO^-^ or H_2_O_2_) were tested by LC-MS. As depicted in **Figure 4A**, large amount of BE was released in H_2_O_2_, while no drug was detected in the other four ROS media or PBS. Furthermore, the effective BE release was observed in 0.25, 0.5 and 1.0 mM H_2_O_2_, while only limited drug release occurred in 0.125 mM H_2_O_2_ and PBS. Especially in 1.0 mM H_2_O_2_, the release ratio of BE was close to 50 % after 30 min according to the BE loading content (25.5%). After 2 h, BE was almost completely released from cPBA-BE (**Figure 4B**). Therefore, the BE release from cPBA-BE showed a H_2_O_2_ concentration dependence. Meanwhile, the drug release in PBS at different pH was also examined. As shown in **Figure 4C**, significant BE release was occurred at pH 5.6 and 6.4, while no drug was released in neutral and alkaline media. The more acidic the environment is, the faster drug release rate appeared. Concretely, nearly 100 % BE was released in pH 5.4 PBS after 30 min. In pH 6.4 PBS, almost all BE released from cPBA-BE needs about 45 mins.

A well-known substance in blood with *o*-diol structures is glucose, which has been previously reported to form ester bonds with PBA derivatives 65, 66. When cPBA-BE was given intravenously, does blood glucose competitively combine with cPBA? As can be seen from **Figure 4D**, the normal level of glucose (6 mmol/L) neither caused any BE release from cPBA-BE, nor hindered the drug release induced by 1 mM H_2_O_2_. This is probably because the concentration of glucose is not enough for significant exchange reaction, and also the boric acid ester bonds in cPBA-BE were located in the hydrophobic core of nanoprodrug, while the outer dense cyclodextrin structure obstructed the contact between glucose and PBA. Similarly, prolonged incubation with 600 mM NaCl neither induced any release of BE, nor hindered the H_2_O_2_-activated drug release from cPBA-BE (**Figure S2**). In summary, cPBA-BE was determined to be a dual-responsive self-assembled nanoprodrug for passive-targeted delivery of BE.

### Internalization and drug release of cPBA-BE nanoprodrug in HepaRG cells

Before investigating the uptake and drug release characteristics of cPBA-BE nanoprodrug in cells, the safety of each component and nanoprodrug were examined to RAW264.7 and HepaRG cells. No matter for BE, cPBA or cPBA-BE, as the concentration increases, the cell viability gradually decreases (**Figure S3**). Although the cytotoxicity of nanoprodrug is a little bit higher than that of BE and cPBA, the viability of both cells was above 85 % even at high concentration of 1000 μg/mL. The results revealed that cPBA-BE nanoprodrug exhibited no significant toxicity to both RAW264.7 and HepaRG cells.

**Figure 5:**
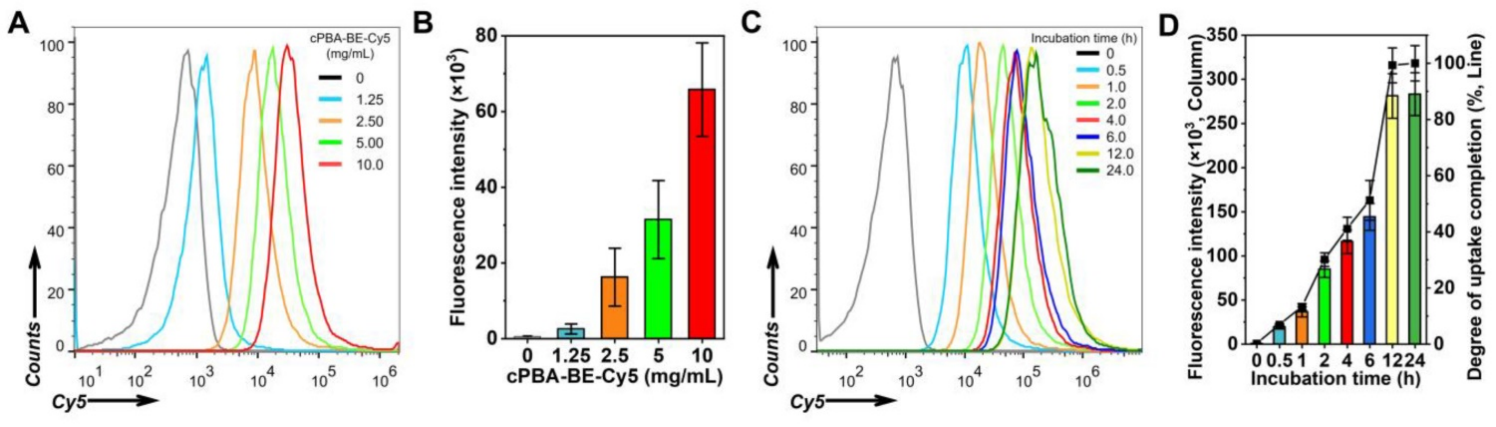
The uptake profiles of cPBA-BE in HepaRG cells. **A-B**, Representative flow cytometry curves (A) and quantitative data (B) illustrating concentration-dependent internalization of Cy5-labled cPBA-BE in HepaRG cells for 2 h. **C-D**, Representative flow cytometry curves (C) and quantitative data (D) illustrating time-dependent internalization of 10 mg/mL Cy5-labled cPBA-BE in HepaRG cells till 24 h. All data are presented as mean ± SD (n = 6).

Flow cytometry was performed to investigate the internalization behavior of cPBA-BE in HepaRG cells. Fluorophore-labeled cPBA-BE was prepared, and both Cy5-labeled and Cy7.5-labeled cPBA-BE showed regular spherical shape, indicated that the Cy dyes didn't affect the self-assembly process of cPBA-BE (**Figure S4**). In dose-dependent experiments, flow cytometry analysis showed enhanced endocytosis of cPBA-BE-Cy5 in HepaRG cells with increased dose (**Figure 5A-B**). With incubation time was prolonged, internalized cPBA-BE-Cy5 notably increased (**Figure 5C-D**). The uptake of cPBA-BE-Cy5 by cells reached the maximum at 12 h. Based on this, the degree of uptake completion at each time point was calculated and exhibited in** Figure 5D**.

In addition, it is important to explore whether internalization destructed cPBA-BE nanostructure and resulted in BE release. LC-MS was used to detect the intracellular BE content after HepaRG cells incubated with 80 μg/mL cPBA-BE for different time. As shown in **Figure S5**, a small amount of BE was leaked after cPBA-BE contacted with cells for 0.5 h, with the release ratio of 4.0 ± 0.38%. As incubation time was prolonged, no further significant leakage was observed. These results suggested that cPBA-BE was internalized by HepaRG cells in a dose- and time-dependent manner. During the internalization process, cPBA-BE nanostructure keep stable without obvious leakage of BE.

### Biological activities of cPBA-BE in APAP-injured HepaRG cells

**Figure 6:**
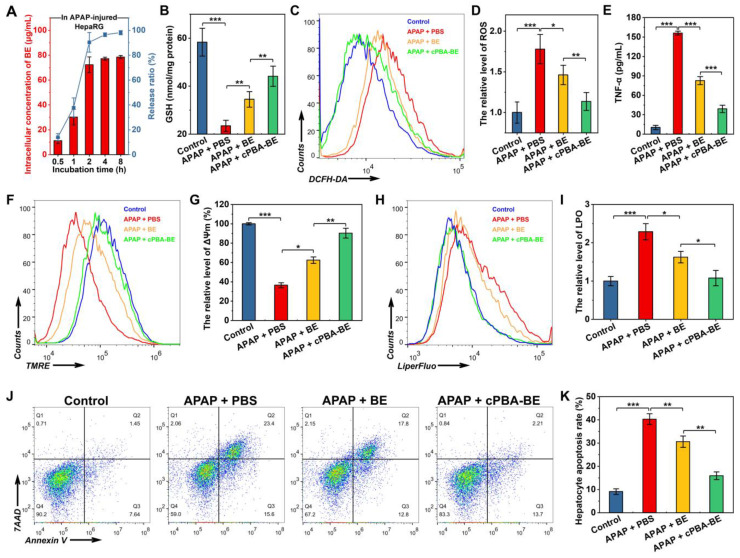
*In vitro* anti-oxidative, anti-inflammatory and anti-apoptosis activity of cPBA-BE in APAP-injured HepaRG cells. HepaRG cells were cultured with 3 mg/mL of APAP, 80 μg/mL cPBA-BE and 20 μg/mL BE were as treatment groups. **A**, Intracellular drug release of cPBA-BE in HepaRG cells injured by 3 mg/mL APAP. **B**, The content of GSH in APAP-injured HepaRG cells determined by kits. **C-D**, Typical flow cytometry profiles (C) and quantitative analysis (D) showing the intracellular generation of ROS in HepaRG cells. DCFH-DA was used as intracellular ROS probe. **E**, The expression level of TNF-α in APAP-injured HepaRG cells. **F-G**, Flow cytometric profiles (F) and quantitative analysis (G) illustrating disruption of mitochondrial membrane potential (ΔΨm). **H-I**, Representative flow cytometry curves (H) and quantitative data (I) of LPO degree in APAP-injured HepaRG cells. **J-K**, Typical flow cytometry (J) and quantitative data (K) of HepaRG cell apoptosis. All data are presented as mean ± SD (n = 6). *p < 0.05, **p < 0.01 and ***p < 0.001; ns, no significance.

Due to HepaRG cells have complete cytochrome oxidase system, it is more suitable for testing drug activities than other cell lines 46, 47. Injured by APAP would significantly increase the level of ROS in hepatocyte-like HepaRG cells 67, and cause the accumulation of endogenous lactic acid, resulting in metabolic acidosis 68. In this study, after HepaRG cells were injured by 3 mg/mL APAP, 80 μg/mL cPBA-BE was added and drug release was detected using LC-MS. Unlike the stability and low drug release of cPBA-BE in normal HepaRG cells (**Figure S5**), the concentration of BE in injured cells is gradually increased as the incubation time extended (**Figure 6A**). The BE has been almost completely released (78.5 ± 1.3 μg/mL, 98.1 ± 1.6%) from cPBA-BE nanoprodrug after 8 h. Such a dramatic difference in normal and APAP-injured HepaRG cells is due to the dual-responsiveness of the cPBA-BE nanoprodrug.

It is generally believed that overdose APAP produces a large number of metabolites through liver CYP450 enzyme metabolism. The metabolites rapidly deplete GSH and cause oxidative stress, resulting in liver cell damage 47. **Figure 6B** shows that APAP significantly depleted GSH in HepaRG cells to 40.2% of that in the control group. Free BE could relieve the depletion of GSH to a certain degree with 59.2% of GSH in the control group, while cPBA-BE shows a better effect to recover the GSH content to 75.6% of that in the control group. In addition, the effects of BE or cPBA-BE on glutathione reductase (GR), which is an important antioxidant enzyme were determined. As shown in **Figure S6,** APAP significantly decreased the GR content to 28.3% of that in the control group, while free BE and cPBA-BE were able to recover it to 57.2% and 79.1%, respectively. Flow cytometry was used to investigate the anti-oxidative activity of cPBA-BE in HepaRG cells with DCFH-DA as ROS probe. It can be seen from **Figure 6C-D,** cultured with 3 mg/mL APAP caused about 1.78-fold increase of the level of ROS in HepaRG cells compared with the control group, whereas treated with 20 μg/mL BE and 80 μg/mL cPBA-BE significantly reduced it to 1.46-fold and 1.13-fold, respectively. Besides, the ability of cPBA-BE treatment to reduce the expression of TNF-α is better than that of free BE treatment, indicating the good anti-inflammatory activity of cPBA-BE nanoprodrug (**Figure 6E**).

Experimental evidences from animal studies, perfused liver slices and cell cultures have shown that the toxic metabolite of APAP, N-acetyl-p-benzo-quinone imine (NAPQI), inhibits electron transfer in the mitochondrial respiratory chain, resulting in severe depolarization of mitochondrial membrane potential (ΔΨm) 69. The effects of different treatments on ΔΨm were quantified by flow cytometry using TMRE as a probe (**Figure 6F**). After APAP injury, the ΔΨm was only 36.5% of that in normal cells. BE treatment and cPBA-BE treatment were able to significantly reverse the depolarization, rising ΔΨm to 62.4% and 90.4% of that in normal cells, respectively (**Figure 6G**). Meanwhile, redox imbalance would significantly affect the degree of lipid peroxidation (LPO) of cells. LiperFluo^™^ was used as a probe to determine the level of LPO by flow cytometry 70. As shown in **Figure 6H-I**, LPO in HepaRG cells increased significantly to 2.29 ± 0.21 times that in normal cells by APAP injury. Both free BE and cPBA-BE treatments inhibit the degree of LPO, while cPBA-BE treatment shows a greater degree of suppression.

In the hepatocyte apoptosis experiment, cells stained with Annexin V (Annexin V^+^/7AAD^-^) were considered to be apoptotic cells, while that stained with 7AAD (Annexin V^+^/7AAD^+^) were considered to be severe necrotic cells. As displayed in **Figure 6J-K**, 3 mg/mL APAP caused apoptosis of HepaRG cells with a rate of nearly 40%. After treated by BE or cPBA-BE, lower hepatocyte apoptosis rates (about 30% for BE treatment and 15% for cPBA-BE treatment) were observed. The protective effect of cPBA on APAP-injured HepaRG cells was further determined by MTT assay (**Figure S7**). Results indicated that cPBA at the concentration less than 200 μg/mL showed no protective effect. As the administrated concentration of cPBA-BE was 80 μg/mL, the cell protective effect of cPBA-BE mainly relied on the local concentration of BE.

Lactate dehydrogenase (LDH) is an important enzyme that would release after liver injury, and its concentration could reflect the apoptosis of liver cells. As can be seen from **Figure S8A**, cPBA-BE significantly reduced the amount of LDH in the culture medium, which is consistent with the results from the apoptosis kit assay. Moreover, aspartate aminotransferase (AST) is another important marker of liver injury, especially in HepaRG cells. The released AST from injured HepaRG cells was dramatically reduced by cPBA-BE, and cPBA-BE is more effective than free BE (**Figure S8B**).

These results indicated that cPBA-BE nanoprodrug can rapidly and fully release BE in APAP-injured HepaRG cells. Compared with free BE treatment, cPBA-BE could better inhibit the GSH depletion, maintain GR activity, relieve the oxidative stress outbreak and lipid peroxidation, and finally resist the HepaRG cell apoptosis.

### *In vivo* biocompatibility of cPBA-BE

The cPBA-BE nanoprodrug was expected to be applied *in vivo*. Prior to evaluating its therapeutic effect, a hemolysis assay was performed at the effective concentration of BE (5 mg/mL). For free BE, 5% DMSO is used as hydrotrope due to the extremely low water-solubility. As shown in**Figure S9,** 20 mg/mL cPBA-BE exhibited a similar low hemolytic behavior as cPBA, which assures the safety of cPBA-BE nanoprodrug for systemic administration. In addition, daily intravenous injection of 100 μL 20 mg/mL cPBA-BE for 12 consecutive days showed high safety to blood and organs, which proved a pre-requisite for potential clinical transformation. Specifically, the daily weight of mice did not change significantly (**Figure S10A**). The number of red blood cells and white blood cells were not affected after the treatment (**Figure S10B-C**). Liver and kidney function indicators represented by AST and BUN showed to be normal (**Figure S10D-E**). H&E stained sections also demonstrated that cPBA-BE didn't cause pathological changes in all major organs (**Figure S11**).

### Pharmacokinetics of cPBA-BE in mice with DIH

**Figure 7:**
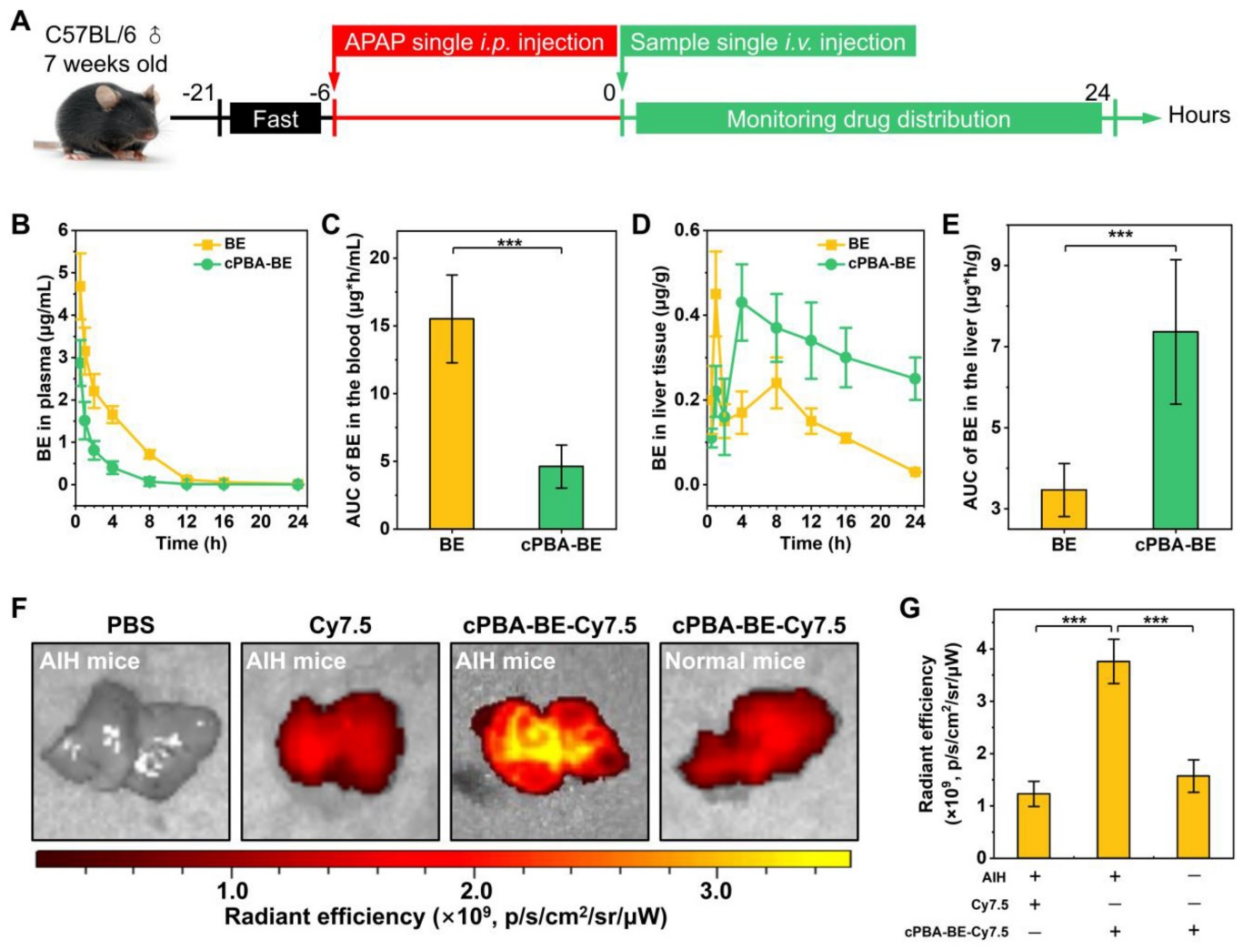
Biodistribution and pharmacokinetics of cPBA-BE in AIH mice. **A**, Scheme of establishment of AIH mice and treatment regimens. **B**, Quantitative analysis of BE concentrations in blood. **C**, Cumulative amount of BE in blood by calculating the area under the curve (AUC). **D-E**, The BE concentrations (D) and cumulative amount of BE (E) in the liver of AIH mice. **F-G**, Representative *ex vivo* images (F) and quantitative analysis of radiation (G) illustrating distribution of Cy7.5 fluorescence signals in liver tissues. In the experiments of Figure B-E, the mice received 20 mg/mL cPBA-BE or 5.1 mg/mL free BE. For Figures F-G, the mice received Cy7.5-labeled cPBA-BE at a dose of 0.5 mg Cy7.5 in each mouse. All data are presented as mean ± SD (n = 3). *p < 0.05, **p < 0.01 and ***p < 0.001; ns, no significance.

It has been proved that the ROS level in liver of the patents with overdose APAP-treatment is several times higher than that in normal people. And inhibition of the respiratory chain and reduction of tissue perfusion after liver necrosis can cause anaerobic metabolism, leading to lactic acid accumulation. Thus, the ROS- and acidic-responsive cPBA-BE nanoprodrug was expected to achieve passive targeting to liver tissue in DIH mice. According to **Figure 7A**, the pharmacokinetics and distribution profiles of cPBA-BE in liver were investigated in mice with APAP-induced hepatitis (AIH).

After 6 h of 200 mg/kg APAP intraperitoneal injection, a single intravenous injection of 100 μL 20 mg/mL cPBA-BE was performed, and the BE content in blood was monitored within 24 h. cPBA-BE treatment could significantly reduce the *C*_max_ of BE. At 0.5 h, the *C*_max_ of BE in BE treatment group was 4.68 ± 0.78 μg/mL, while the *C*_max_ of BE released from cPBA-BE was 2.87 ± 0.54 μg/mL, and thereafter decreased with time (**Figure 7B**). Correspondingly, by calculating the AUC, cPBA-BE treatment significantly reduced the cumulative amount of BE in blood (**Figure 7C**). However, the pharmacokinetic characteristics of free BE and cPBA-BE in liver tissue were quite different. Although cPBA-BE treatment had no significant effect on the *C*_max_ of BE, it extended the time to reach *C*_max_ (*T*_max_) from 1 h to 4 h (**Figure 7D**). The concentration of BE in free BE group reached the peak quickly, then the drug-time curve showed a rapid downward trend. But in cPBA-BE group, the concentration of BE gradually increased to a peak, and then decreased much more slowly. The reason may be that nanoprodrug form changed the primary distribution characteristics of BE. Moreover, the cumulative amount of BE in liver in cPBA-BE group was 2.1-times higher than that in free BE group (**Figure 7E**), which is resulted from the liver targeting and responsive drug release of cPBA-BE.

E*x vivo* imaging experiment further confirmed the hepatitis targeting of cPBA-BE. Specifically, Cy7.5-labeled cPBA-BE (cPBA-BE-Cy7.5) was injected intravenously, and the liver was collected and imaged after 24 h. As shown in **Figure 7F-G**, cPBA-BE-Cy7.5 showed a much stronger fluorescence signal in the liver of AIH mice than that in the liver of normal mice, and the fluorescence intensity was nearly 3 times of that in free Cy7.5 group. Besides, the fluorescence signal of cPBA-BE-Cy7.5 in other major organs of AIH mice is much lower than that in liver (**Figure S12**). These results suggested that cPBA-BE tends to specifically accumulate to the injured liver tissue.

### cPBA-BE alleviate APAP-induced acute hepatitis

**Figure 8:**
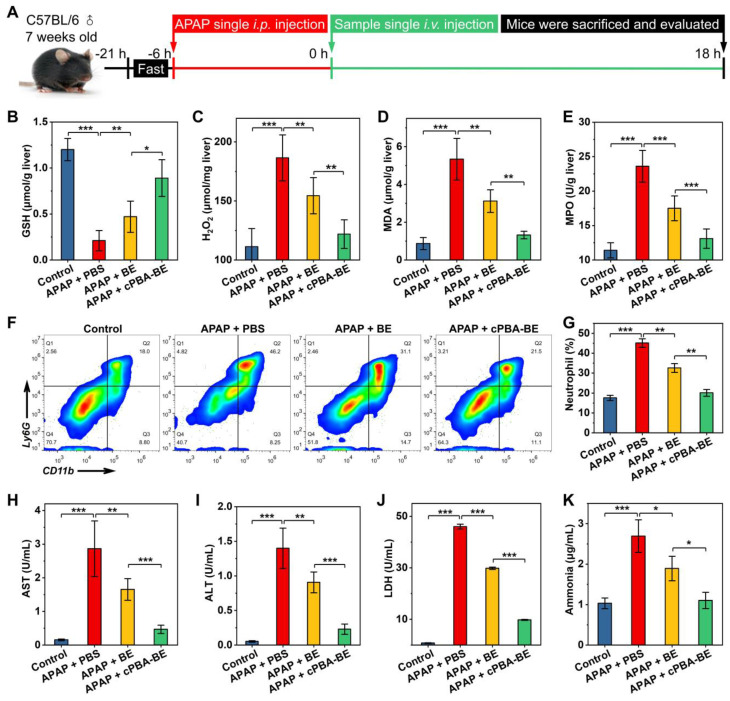
*In vivo* efficacy of cPBA-BE for alleviating APAP-induced acute hepatitis. **A**, Establishment of AIH mice and treatment regimens. After mice injured by 200 mg/kg APAP for 6 h, single intravenous injection of 100 μL 20 mg/mL cPBA-BE in PBS or 5.1 mg/mL BE in 5% DMSO (equal to 20 mg/mL cPBA-BE) was performed. **B-E**, The expression levels of representative factors of inflammation and oxidative stress in liver. After 18 h of treatment, homogenates of the hepatic tissues were prepared, and the concentrations of GSH (B), H_2_O_2_ (C), MDA (D) and MPO (E) were separately measured. **F-G**, Representative flow cytometric analysis (F) and quantitative analysis (G) of the proportion of neutrophils in liver tissues. **H-K**. The AST (H), ALT (I), LDH (J), ammonia (K) levels in serum. All data are presented as mean ± SD (n = 5). *p < 0.05, **p < 0.01 and ***p < 0.001; ns, no significance.

The alleviation effect of cPBA-BE on APAP-induced acute hepatitis were investigated according to **Figure 8A**. After 6 h of single *i.p.* injection of 200 mg/kg APAP, mice were *i.v.* injected with 100 μL 20 mg/mL cPBA-BE or 5.1 mg/mL BE. As shown in **Figure 8B**, the GSH concentration of normal liver tissue was 1.2 ± 0.12 μmol/g. After excessive APAP injury, GSH was almost depleted (0.21 ± 0.11 μmol/g). BE can act as an activator of Nrf-2 pathway and up-regulate the antioxidant enzyme system, thereby antagonize the toxicity of APAP 25. Therefore, cPBA-BE nanoprodrug with high BE release can dramatically increase GSH content (0.89 ± 0.2 μmol/g), while BE only recover it to 0.47 ± 0.17 μmol/g. Due to the down regulation of the antioxidant, an oxidative stress bursts in liver tissue after acute injury. Treated by cPBA-BE and BE significantly reduced the level of H_2_O_2_ to 65.3% and 82.8% of that in APAP-injured group, respectively (**Figure 8C**). Similarly, cPBA-BE down regulates MDA and MPO more observably than free BE (**Figure 8D-E**), suggesting that cPBA-BE alleviates the oxidative stress and tissue damage induced by APAP more effectively. It is generally known that MPO is a specific marker of neutrophils, and believed that neutrophils also played a key role in the occurrence and development of acute DIH 71. The flow cytometry and quantitative analysis showed that the proportion of neutrophils (CD11b^+^Ly6G^+^) in lymphocytes of AIH mice liver raised to about 45.1 ± 2.2 % (**Figure 8F-G**). Although BE can reduce the recruitment of neutrophils to a certain extent (32.6 ± 2.2 %), cPBA-BE (20.1 ± 1.7 %) acts more effectively to recover it close to normal level (17.6 ± 1.3 %).

Meanwhile, the expression levels of two important inflammatory factors (TNF-α and IL-1β) in liver tissues were measured. As shown in **Figure S13**, expression of TNF-α and IL-1β in AIH mice were 1.69 times and 1.44 times higher than those in normal mice, respectively. cPBA-BE could significantly decrease the levels of TNF-α and IL-1β to 1.13 times and 1.07 times of that in normal mice. This revealed the excellent anti-inflammatory effect of cPBA-BE.

The concentrations of important hematological indicators (AST, ALT, LDH and ammonia) were determined and shown in **Figure 8H-K**. Normally, the serum AST and ALT content is low. When the hepatocytes are damaged, the increased cell membrane permeability allows AST and ALT in the cytoplasm released into the circulation, resulting in the serum concentration of both biomarkers significantly increased 72. Here, serum AST and ALT in AIH mice were 18.5 and 26.6 times of that in normal mice, respectively (**Figures 8H-I**). And AST/ALT > 1 in AIH mice suggested severe liver damage. After intervention with cPBA-BE, serum AST and ALT decreased by 83.7 % and 83.6 %, respectively, which was significantly more effective than free BE. Likewise, both serum LDH and ammonia increased significantly after liver damage, and cPBA-BE reduced them more effectively than free BE (**Figure 8J-K**). Especially, the serum ammonia level in cPBA-BE group almost recover to normal level.

**Figure 9:**
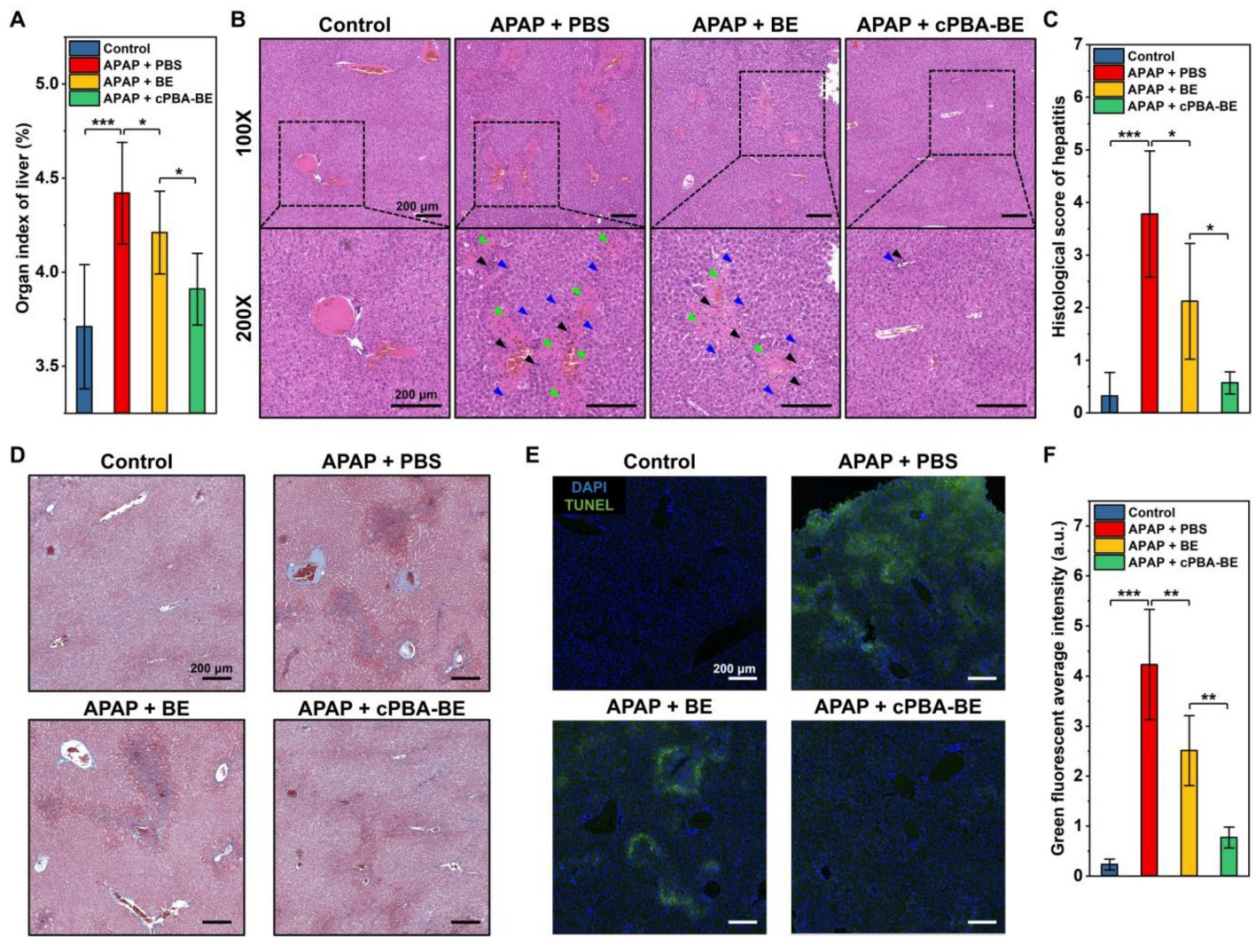
Histological evaluation of APAP-induced acute hepatitis. **A**, Organ index of liver in AIH mice. **B**, H&E-stained histological sections of hepatic tissues from AIH mice with or without various treatments at two magnifications (100× and 200×). Black, blue, and green arrows indicate immune cell infiltration, hepatocyte dilation, and necrosis area, respectively. **C**, Histological scores of hepatitis in mice. **D**, Masson trichrome (MT) staining of liver sections. **E-F**, Analysis of hepatic cell apoptosis by TUNEL assay. All scale bar represents 200 μm. All data are presented as mean ± SD. For A, n = 5, for C and F, n = 10. *p < 0.05, **p < 0.01 and ***p < 0.001; ns, no significance.

The histological evaluations were further performed. **Figure 9A** shows that APAP causes an increase in the organ index of liver, indicating the edema was occurred. Treatment with cPBA-BE clearly decreased the organ index of liver more obviously than free BE. Additionally, histological examination revealed considerable necrotic cell death of hepatocytes, cell vacuolization, significant injury/necrosis of sinusoidal endothelial cells, as well as local hemorrhage in the model group. Whereas the free BE treatment only slightly suppressed the necrosis area and damage in AIH mice, a more significant effect was attained by treatment with the same dose of cPBA-BE (**Figure 9B**). Correspondingly, histological score of liver damage was also evaluated as previous report 73. The histological score of sections from the mice treated with cPBA-BE was comparable to that from the normal mice treated with PBS (**Figure 9C**). Masson staining also suggested that cPBA-BE could significantly alleviate liver fibrosis caused by APAP (**Figure 9D**).

Furthermore, the degree of apoptosis of hepatocytes was evaluated by TUNEL staining. In **Figure 9E-F**, green fluorescence represents apoptotic or necrotic liver cells. APAP primarily damaged the cells around hepatic lobular vein to form annular necrosis, then continually injured other liver parenchyma, resulting in extensive cell apoptosis. Although BE could alleviate the apoptosis of liver parenchymal cells to a certain extent, complete repair of damaged cells around the vein cannot be achieved. In contrast, cPBA-BE showed a better alleviation and repair effect on apoptosis of liver cells because of the targeted distribution of cPBA-BE and local high concentration of BE. In addition, overdose APAP is also reported to cause some degree of nephrotoxicity 74. As revealed in **Figure S14A**, treatment with cPBA-BE effectively alleviated hydronephrosis. Combined with the reduction of CREA and UREA in cPBA-BE group (**Figure S14B-C**), cPBA-BE was proved to be effective to mitigate the nephrotoxicity associated with AIH. Given the above, cPBA-BE has significantly better efficacy than free BE to alleviate APAP-induced acute hepatitis.

### cPBA-BE alleviate RFP-induced chronic hepatitis

**Figure 10:**
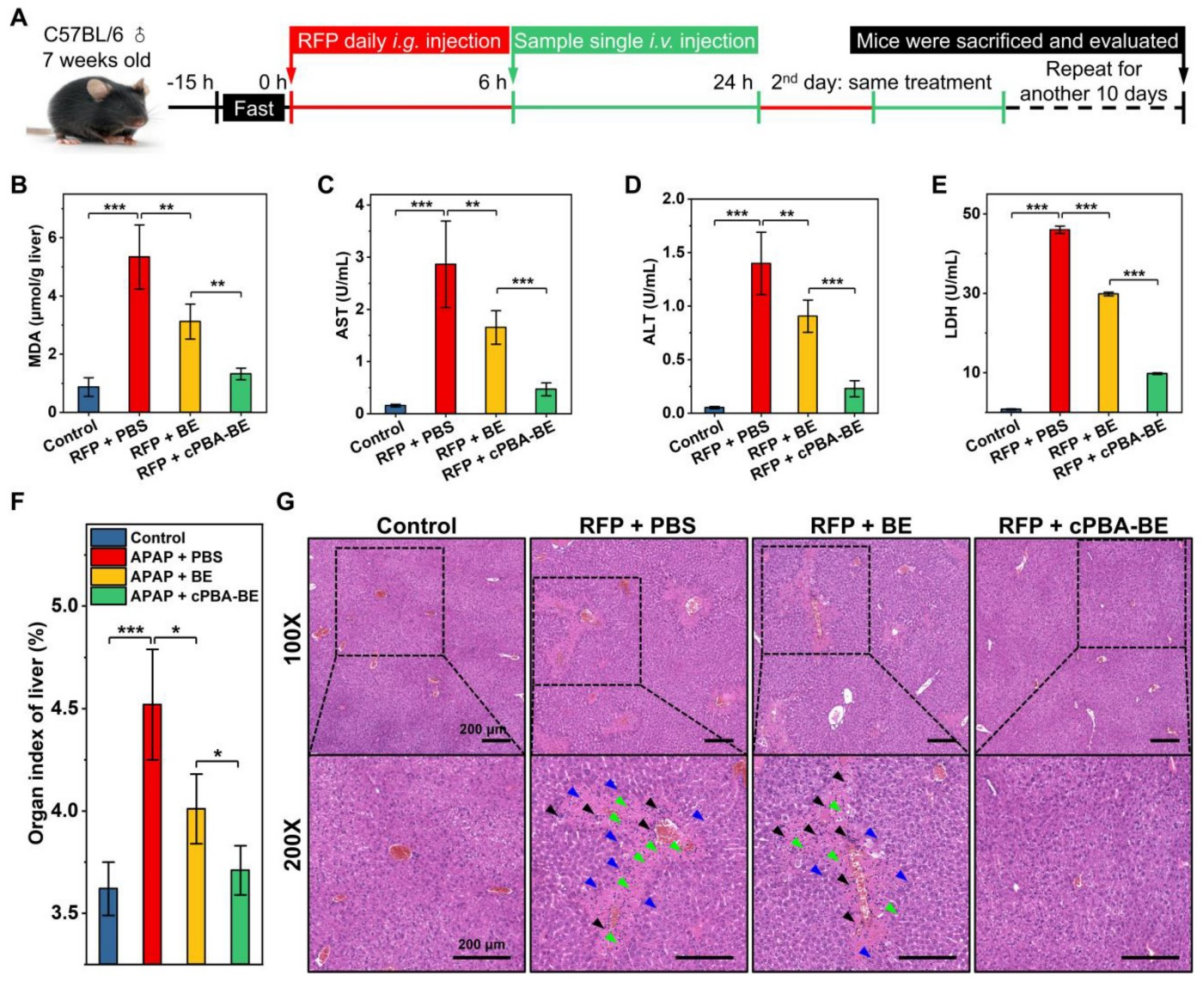
Therapeutic effect of cPBA-BE on RFP-induced chronic hepatitis. **A**, Establishment of RIH mice and treatment regimens. After oral administration of RFP for 6 h, single intravenous injection of 100 μL 20 mg/mL cPBA-BE in PBS or 5.1 mg/mL BE in 5% DMSO was performed. This process lasted 12 days. **B**, The MDA content in liver reflecting the degree of lipid peroxidation. **C-E**, The AST (C), ALT (D) and LDH (E) levels in plasma. **F**, Organ index of liver in RIH mice. All data are presented as mean ± SD (n = 5). **G**, H&E-stained histological sections of hepatic tissues from RIH mice with or without various treatments. The magnification is 100 and 200 times. Black, blue, and green arrows indicate immune cell infiltration, hepatocyte dilation, and necrosis area, respectively. *p < 0.05, **p < 0.01 and ***p < 0.001; ns, no significance.

Chronic hepatitis is another noteworthy side effect of long-term use of drugs in clinic. For instance, patients with tuberculosis need to take anti-tuberculosis drugs for a long time, which causes typical rifampin-induced chronic hepatitis (RIH). To evaluate the effect of cPBA-BE on RFP-induced chronic hepatitis, the experimental protocol was designed according to the previous report 51. Chronic hepatitis model was established by orally administering 200 mg/kg rifampin (RFP) daily for 12 consecutive days. After 6 h of RFP administration every day, cPBA-BE or free BE was given intravenously, with PBS treatment as a control group (**Figure 10A**). Within 12 days, administration of RFP led to significantly less food intake and a slow weight loss. BE and cPBA-BE treatments showed certain positive effects on the maintenance of body weight (**Figure S15A**). Mice were sacrificed on the 12^th^ day, blood samples and liver tissues were collected for hematological analysis and histological evaluation, respectively. Similar to the results obtained in the AIH model, cPBA-BE treatment alleviated oxidative stress as the MDA level in liver was down-regulated (**Figure 10B**).

Compared with the model group, serum AST, ALT and LDH in cPBA-BE treatment group all decreased more obviously than that in BE treatment group (**Figure 10C-E**), indicating cPBA-BE exhibits superior effect than free BE on restoring liver function and resisting hepatocyte necrosis. It is known that long-term use of RFP can increase the organ index of the liver due to cholestatic hepatitis. From** Figure 10F**, cPBA-BE shows a better mitigating effect on edema than free BE. Moreover, cPBA-BE effectively reduced hepatocyte necrosis, chronic inflammatory cell infiltration, and tissue damage caused by RFP according to the histological evaluation (**Figure 10G**).

Meanwhile, the expression levels of TNF-α and IL-1β in liver of RIH mice were 2.42 times and 2.20 times of that in liver of normal mice, respectively (**Figure S15B-C**). cPBA-BE could significantly reduce the levels of TNF-α and IL-1β to 1.20 times and 1.30 times of that in normal mice. This revealed the excellent anti-inflammatory effect of cPBA-BE on RIH. To sum up, the above results indicated that cPBA-BE has good efficacy on alleviating RFP-induced chronic hepatitis.

## Conclusion

In summary, a stimuli-activated HP-βCD-based circular PBA (cPBA) has been designed and synthesized. ROS- and acid-responsive cPBA-BE nanoprodrug was self-assembled via boronic acid coupling between the hydrophilic cPBA and hydrophobic BE. Thus, the hydrophobic BE with low bioavailability has been successfully transformed to a pharmacologically efficient nanomedicine. cPBA-BE exhibited good biocompatibility and promoted the concentration-dependent and time-dependent uptake by human hepatocytes-like HepaRG cells. Although cPBA-BE can remain stable in normal HepaRG cells, the nanostructure of cPBA-BE would be disintegrated because of the ROS outbreak and acidosis induced by APAP, resulting in rapid and sufficient release of BE in APAP-injured cells. Accordingly, cPBA-BE can significantly alleviate GSH depletion, oxidative stress, depolarization of mitochondrial membrane potential caused by APAP, and down-regulate the LPO level of cell, thereby reduce HepaRG cell apoptosis. *In vivo* drug distribution and *ex vivo* imaging have shown that cPBA-BE can decrease the concentration of BE in the blood to avoid non-specific distribution, while specifically increasing the concentration in the liver tissues injured by APAP. In the APAP-induced acute hepatitis model and RFP-induced chronic hepatitis model, cPBA-BE could resist GSH depletion, reduce oxidative stress, block the infiltration of neutrophils, and lower the secretion of inflammatory cytokines TNF-α and IL-1β more effectively than free BE. In addition, cPBA-BE exhibits better efficacy on alleviating the acute and chronic DIH than free BE. Specifically, cPBA-BE could reverse the content of hepatitis biomarkers such as serum AST, ALT, LDH and ammonia, and relieve the symptoms of edema, hemorrhage, and cell necrosis in liver tissues. Besides, cPBA-BE also effectively alleviated the nephrotoxicity caused by APAP. In conclusion, cPBA deserves further development as a good platform to couple with hydrophobic drugs containing *o*-diol moieties to prepare nanoprodrug for specific treatment of inflammation-related diseases.

## Supplementary Material

Supplementary figures.

## Abbreviations

7AAD: 7-amino-actinomycin D
AIH: acetaminophen-induced hepatitis
ALT: alanine aminotransferase
APAP: acetaminophen
AST: aspartate aminotransferase
AUC: area under the curve
BE: baicalein
BUN: blood urea nitrogen
CAC: critical aggregation concentration
CDI: N,N'-carbonyldiimidazole
CREA: creatinine
Cy: cyanine
CYP450: cytochrome P450
DAPI: 4',6-diamidino-2-phenylindole
DCM: dichloromethane
DIH: drug-induced hepatitis
DLC: drug loading content
DLS: dynamic light scattering
DMEM: dulbecco's modified eagle medium
DMF: N,N-dimethyllformamide
DMSO: dimethyl sulfoxide
DPBS: dulbecco's phosphate buffered saline
FBS: fetal bovine serum
GR: glutathione reductase
GSH: glutathione
HBSS: Hank's balanced salt solution
H&E: hematoxylin & eosin
HMP: 4-hydroxymethyl-phenol
HP-βCD: hydroxypropyl-β-cyclodextrin
IL-1β: interleukin-1 beta
LC-MS: liquid chromatography mass spectrometry
LDH: lactate dehydrogenase
LPO: lipid peroxides
MDA: malondialdehyde
MPO: myeloperoxidase
MT: masson trichrome
MTT: methyl thiazolyl tetrazolium
NAC: N-acetyl-*L*-cysteine
NAPQI: N-acetyl-p-benzo-quinone imine
Nrf-2: nuclear factor-E2 related factor 2
PBA: phenylboronic acid
PDI: polymer dispersity index
RIH: rifampicin-induced hepatitis
RFP: rifampicin
ROS: reactive oxygen species
TEA: triethylamine
TEM: transmission electron microscope
TMRE: tetramethylrhodamine ethyl ester
TNF-α: tumor necrosis factor alpha
TUNEL: terminal deoxynucleotidyl transferase mediated dUTP nick-end labeling
