# Microenvironment Activatable Nanoprodrug Based on Gripper-like Cyclic Phenylboronic Acid to Precisely and Effectively Alleviate Drug-induced Hepatitis: Erratum

**DOI:** 10.7150/thno.74167

**Published:** 2022-05-28

**Authors:** Qixiong Zhang, Shanshan Li, Lulu Cai, Yuxuan Zhu, Xingmei Duan, Peidu Jiang, Lei Zhong, Kun Guo, Rongsheng Tong

**Affiliations:** 1 Personalized Drug Therapy Key Laboratory of Sichuan Province, Department of Pharmacy, Sichuan Academy of Medical Science & Sichuan Provincial People's Hospital, School of Medicine, University of Electronic Science and Technology of China, Chengdu 610072, China; 2 College of Pharmacy, Southwest Minzu University, Chengdu 610000, China

In the original publication, Figure 8D/H/I/J and Figure 10B/C/D/E showed the MDA, AST, ALT, and LDH levels in APAP-induced acute hepatitis model and RFP-induced chronic hepatitis model, respectively. We are sorry that Figure 10B/C/D/E were presented the same as Figure 8D/H/I/J by mistake. The corrected Figure 10B/C/D/E are shown below. The authors confirm that the correction would not change the result and conclusion of this article. The authors sincerely apologize for any inconvenience or misunderstanding that may have caused.

## Figures and Tables

**Figure 1:**
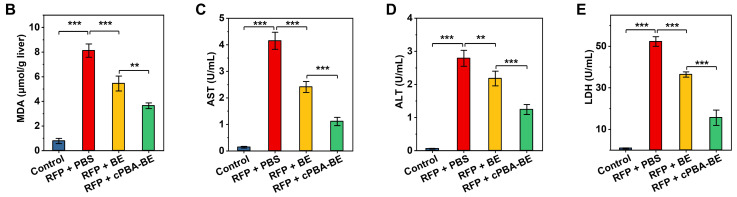
Corrected histograms for the original Figure 10B/C/D/E.

