# Supplementary material for: Microenvironment Activatable Nanoprodrug Based on Gripper-like Cyclic Phenylboronic Acid to Precisely and Effectively Alleviate Drug-induced Hepatitis
Source: Theranostics. 2021 Jul 13;11(17):8301–21. doi: 10.7150/thno.61214 (PMC8344015; doi:10.7150/thno.61214)
Supplement: Supplementary file 1 — Supplementary figures. [file thnov11p8301s1.pdf]

Supplementary Materials

Microenvironment Activatable Nanoprodrug Based on Gripper-like Cyclic Phenylboronic Acid to Precisely and Effectively Alleviate Drug-induced Hepatitis

Qixiong Zhang [1, *, a], Shanshan Li [2, a], Lulu Cai [1], Yuxuan Zhu [1], Xingmei Duan [1], Peidu Jiang [1], Lei Zhong [1], Kun Guo [2], Rongsheng Tong [1, *]

1. Personalized Drug Therapy Key Laboratory of Sichuan Province, Department of Pharmacy, Sichuan Academy of Medical Science & Sichuan Provincial People's Hospital, School of Medicine, University of Electronic Science and Technology of China, Chengdu 610072, China

2. College of Pharmacy, Southwest Minzu University, Chengdu 610000, China

a. These authors contributed equally to this work.

*Corresponding authors:

Qixiong Zhang, PhD, Prof.,

Personalized Drug Therapy Key Laboratory of Sichuan Province, Department of Pharmacy, Sichuan Academy of Medical Science & Sichuan Provincial People's Hospital, School of Medicine, University of Electronic Science and Technology of China

E-mail: qixiongzhang@outlook.com, zhangqixiong@med.uestc.edu.cn (Qixiong Zhang)

*Corresponding authors:

Rongsheng Tong, PhD, Prof.,

Personalized Drug Therapy Key Laboratory of Sichuan Province, Department of Pharmacy, Sichuan Academy of Medical Science & Sichuan Provincial People's Hospital, School of Medicine, University of Electronic Science and Technology of China

E-mail: 2207132448@qq.com (Rongsheng Tong)

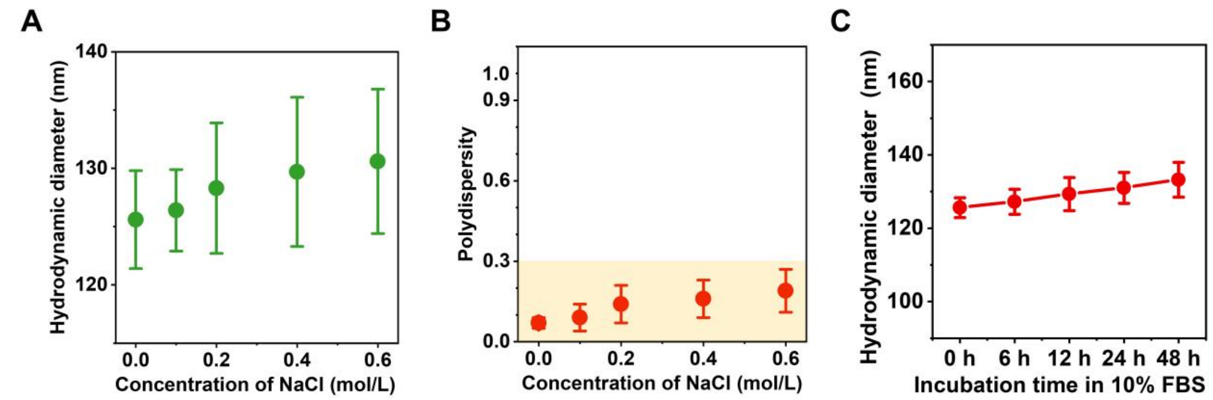

Figure S1. Hydrodynamic diameter (**A**) and polydispersity (**B**) of cPBA-BE at different concentrations (0–0.6 mol/L) of NaCl. Hydrodynamic diameter of cPBA-BE in the medium with 10% FBS at different incubation time points **(C)**. All data are presented as mean ± SD (n = 6).

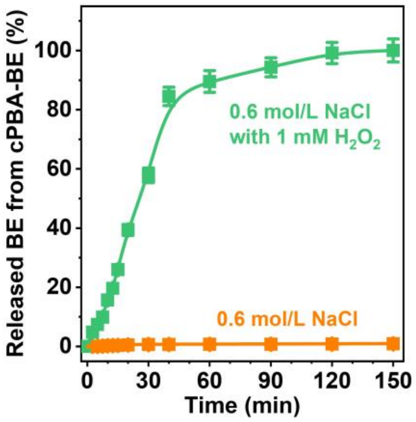

Figure S2. Drug release profiles of cPBA-BE at 0.6 mol/L NaCl with or without 1 mM H_2O_2. All data are presented as mean ± SD (n = 6).

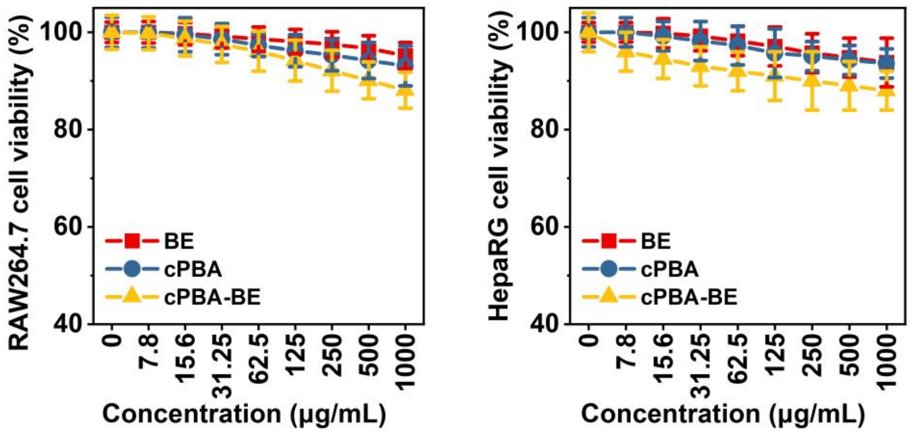

Figure S3. Toxicity of BE, cPBA and cPBA-BE. MTT proliferation assay in RAW264.7 cells and HepaRG cells. All data are presented as mean ± SD (n = 6).

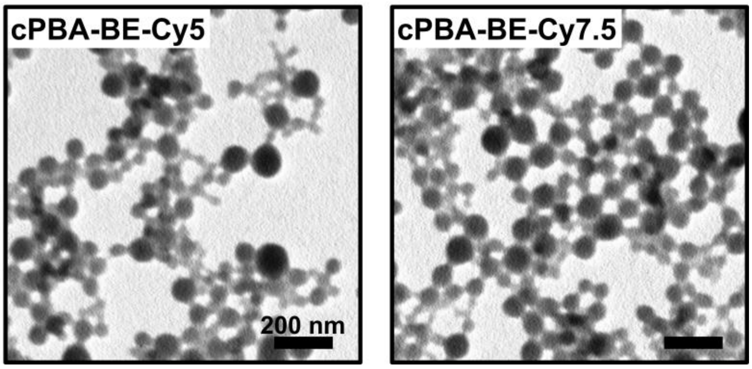

Figure S4. TEM images of cPBA-BE-Cy5 and cPBA-BE-Cy7.5.

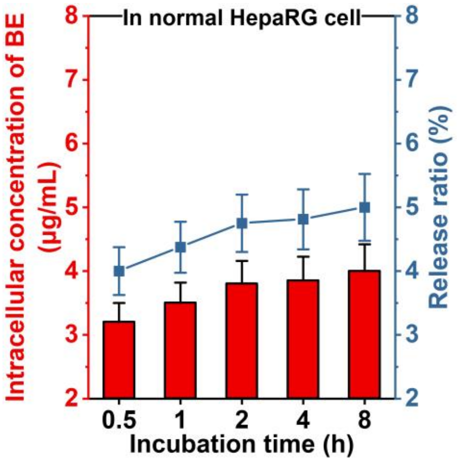

Figure S5. The intracellular concentration of BE and drug release ratio of 80 μg/mL cPBA-BE in normal HepaRG cells at different time. Data are presented as mean ± SD (n = 6).

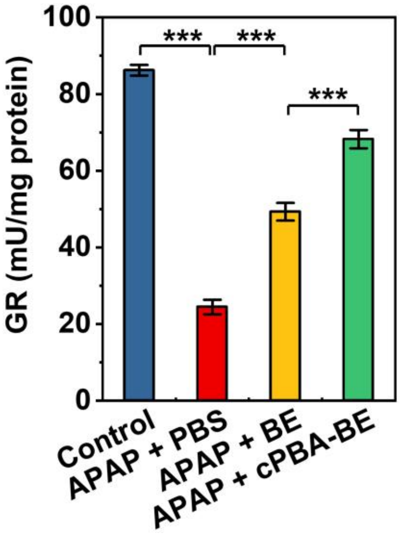

Figure S6. The content of glutathione reductase (GR) in APAP-injured HepaRG cells determined by kits. All data are presented as mean ± SD (n = 6). ***p < 0.001.

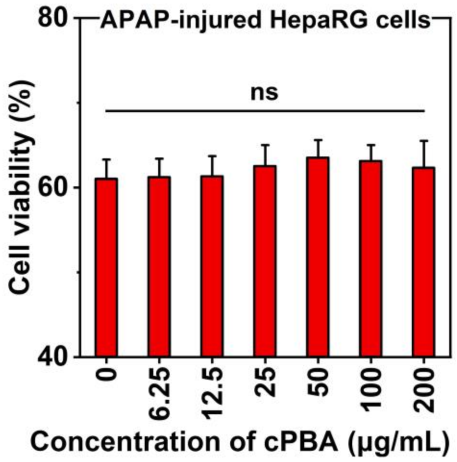

Figure S7. Cell viability of APAP-injured HepaRG cells after incubation with different concentrations of cPBA. All data are presented as mean ± SD (n = 6). ns means no significance.

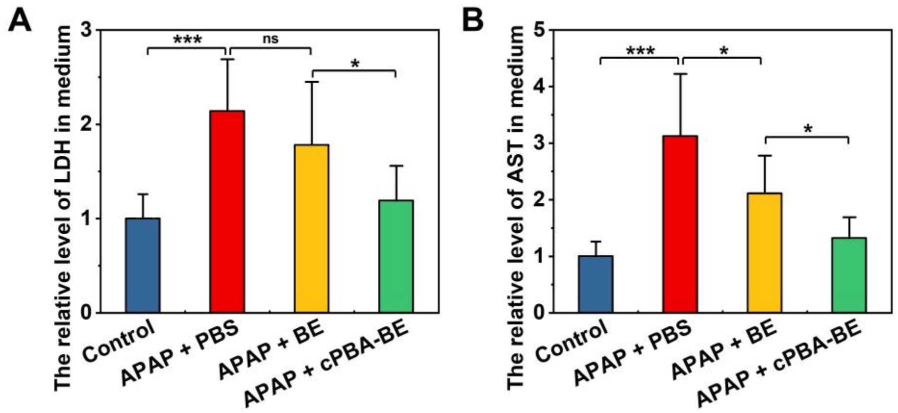

Figure S8. The level of biomarkers of APAP-induced injury in HepaRG cells. **A-B**, The relative level of LDH (A) and AST (B) in culture medium. All data are presented as mean ± SD (n = 5). *p < 0.05, ***p < 0.001; ns, no significance.

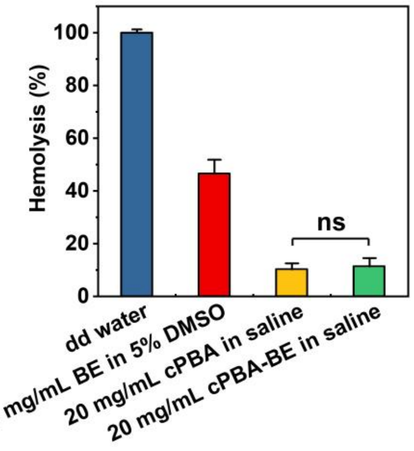

Figure S9. Hemolysis rates of 5 mg/mL BE in 5% DMSO, 20 mg/mL cPBA in saline and 20 mg/mL cPBA-BE in saline. All data are presented as mean ± SD (n = 6). ns, no significance.

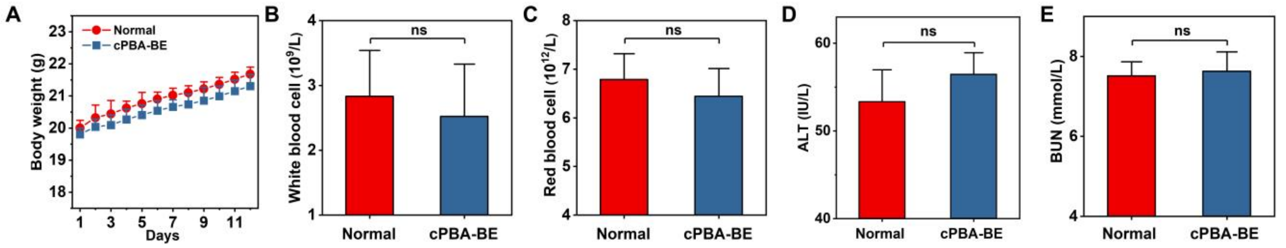

Figure S10. Mice were daily administrated 100 μL 20 mg/mL cPBA-BE for 12 days by intravenous injection. **A**, Body weight changes of the mice during 12 days. **B-C**, Typical hematological parameters including white blood cell and red blood cell on day 12. **D-E**, Levels of biochemical markers relevant to liver (aspartate aminotransferase, AST) and kidney (blood urea nitrogen, BUN) functions on day 12. All data are presented as mean ± SD (n = 5). ns, no significance.

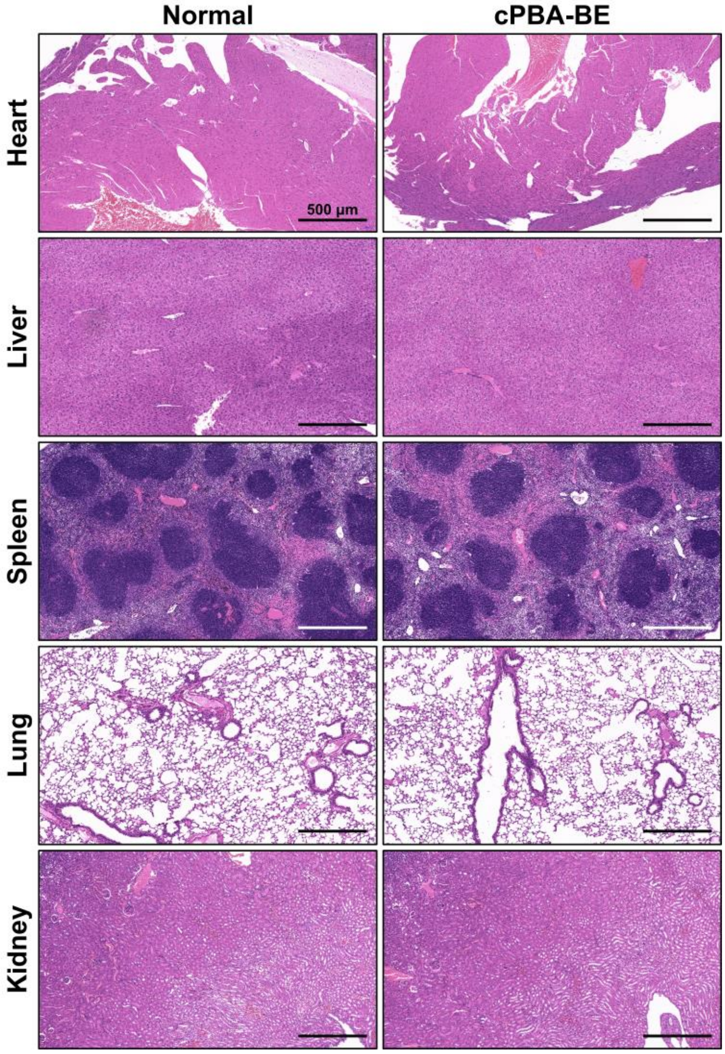

Figure S11. H&E-stained sections of major tissues. Major organs were resected from mice after intravenous administration of 100 μL of cPBA-BE (20 mg/mL) for 12 days. All scale bar represent 500 μm.

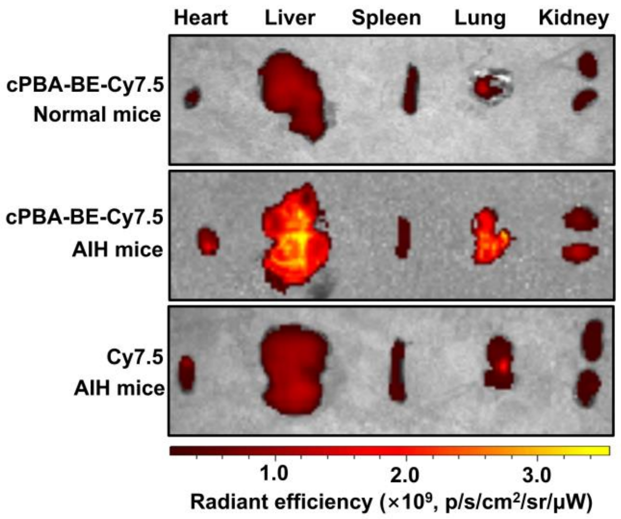

Figure S12. Accumulation of cPBA-BE-Cy7.5 in major organs of AIH mice or normal mice.

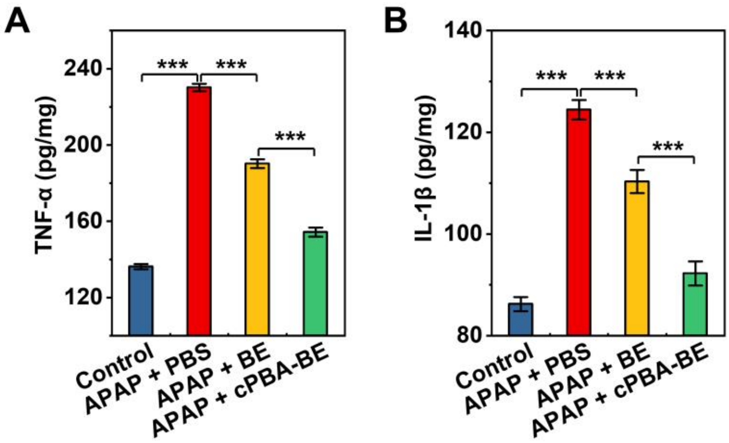

Figure S13. The expression levels of TNF-α (**A**) and IL-1β (**B**) of liver tissues in AIH mice. All data are presented as mean ± SD (n = 5). ***p < 0.001.

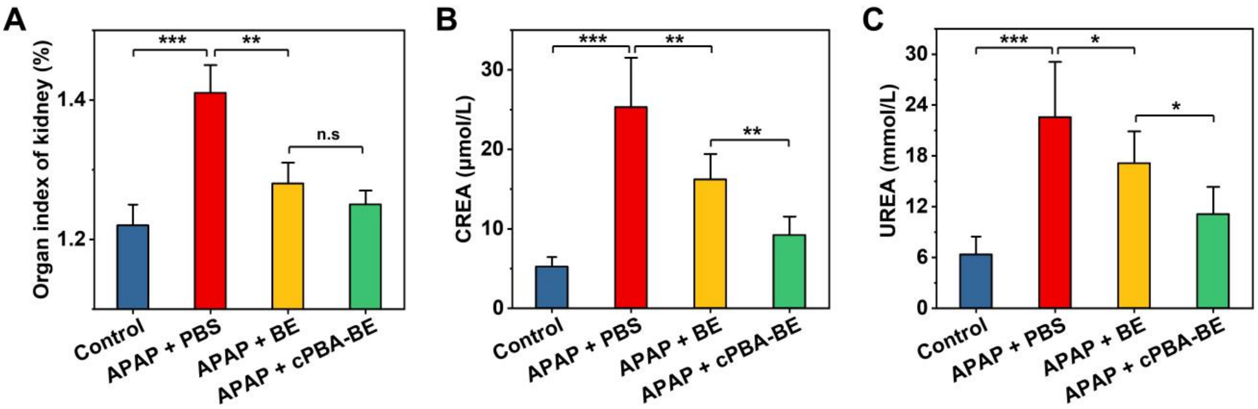

Figure S14. Biomarkers of kidney function in AIH mice. **A**, Organ index of kidney in AIH mice. **B-C**, The concentration of CREA (B) and UREA (C). All data are presented as mean ± SD (n = 5). *p < 0.05, **p < 0.01 and ***p < 0.001; ns, no significance.

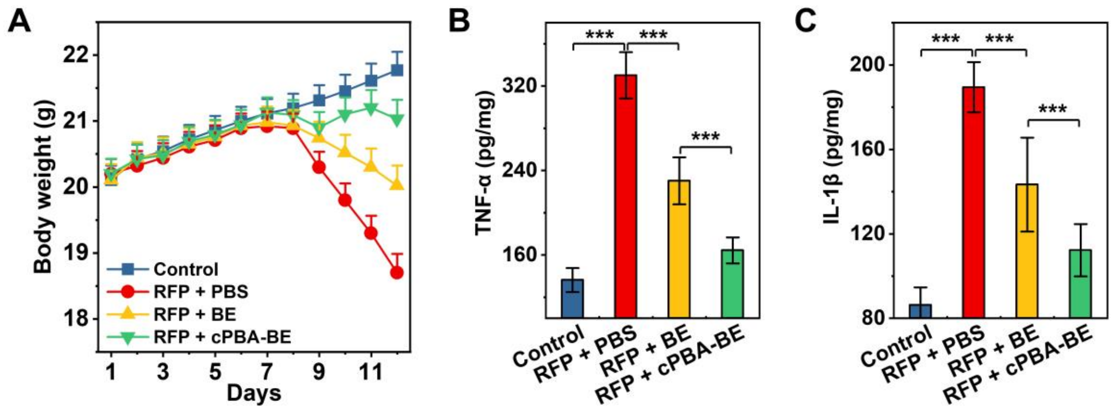

Figure S15. **A**, Body weight changes of RIH mice during 12 days. The expression levels of TNF-α (**B**) and IL-1β (**C**) of liver tissues in RIH mice. All data are presented as mean ± SD (n = 5), ***$p < 0.001$.
